# Spatiotemporal distribution, abundance, diversity and risk of human exposure to phlebotomine sand flies, vectors of *Leishmania* in Belize

**DOI:** 10.1186/s13071-026-07594-9

**Published:** 2026-08-07

**Authors:** Ngwa Niba Rawlings, Mark Stephen Bailey, Orin Courtenay

**Affiliations:** 1 Department of Environmental Health, Defence Medical Services, Ministry of Defence, London, UK; 2 School of Life Sciences, University of Warwick, Coventry, UK; 3 Warwick Medical School, University of Warwick, Coventry, UK; 4 Academic Department of Military Medicine, Royal Centre for Defence Medicine, Birmingham, UK; 5 Zeeman Institute, University of Warwick, Coventry, UK

**Keywords:** Belize, Cutaneous leishmaniasis, Distribution, Diversity, *Leishmania*, Military personnel, Phlebotomine, Sand flies, Spatiotemporal, Vector, Visceral leishmaniasis

## Abstract

**Background:**

Twenty-six sand fly species (Diptera: Psychodidae) are documented to occur in Belize to date, including in regions of human cutaneous leishmaniasis (CL); however, contemporary spatiotemporal compositions of sand flies and CL vectors, and the associated geographical risk of human CL, remain poorly understood.

**Methods:**

Sand flies were sampled in six sentinel sites across a range of ecological zones between April 2023 and May 2025 using CDC light traps, human landing catches, and Biogents Sentinel traps. Specimens were morphologically identified, and spatiotemporal variations in population parameters (abundance, diversity, and vector compositions) analysed using mixed-effects general linearised models. In conjunction with recent human CL case incidence data, a Composite Exposure Risk Index (CERI) was calculated.

**Results:**

A total of 7,759 sand flies representing 20 species and 10 genera were collected over two years sampling, identifying seven known *Leishmania* vectors. Five species, *Dampfomyia deleoni*, *Trichophoromyia triramula*, *Pintomyia ovallesi*, *Psychodopygus panamensis*, and *Brumptomyia mesai*, accounted for > 90% of all captures. Sand fly abundance varied significantly by sampling site and year. Species richness and diversity were highest in intact forest habitats and lowest in coastal and disturbed environments. CERI values identified areas of high to low risk of human-sand fly contact leading to clinical CL. *Lutzomyia longipalpis*, the principal vector of visceral leishmaniasis in the Americas, and *Psathyromyia soccula*, were recorded for the first time in Belize.

**Conclusions:**

This study provides a contemporary quantification of sand flies and sand fly vector spatiotemporal abundance and diversity in north-west and central-east Belize, highlighting areas of higher potential human risk of *Leishmania* exposure.

**Graphical Abstract:**

**Figure:**
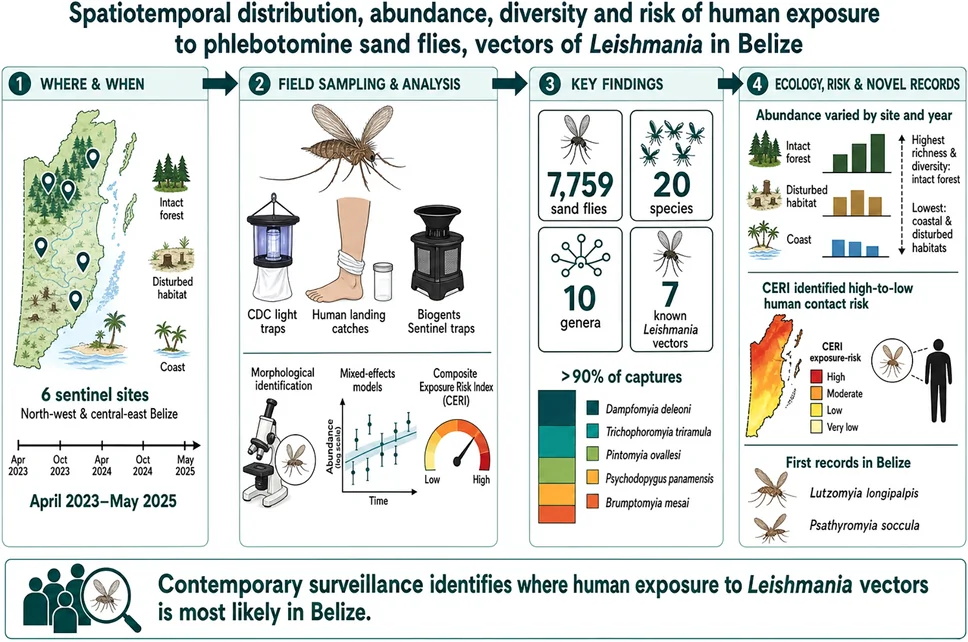

**Supplementary Information:**

The online version contains supplementary material available at https://doi.org/10.1186/s13071-026-07594-9.

## Background

Phlebotomine sand flies (Diptera, Psychodidae, Phlebotominae) are important vectors of human and animal infectious agents including *Leishmania* (Kinetoplastida: Trypanosomatidae) protozoan parasites [1], Phleboviruses [2] and bacteria of the genus *Bartonella* (Rhizobiales: Bartonellaceae) [3, 4]. There are at least 20 species of *Leishmania* that cause human leishmaniasis with an estimated 700,000–1 million annual new cases reported globally [5]. More than 90 sand fly species are recognised as proven or suspected vectors of *Leishmania*, including 56 species in the New World [1]. Many Central and South American countries endemic for leishmaniasis host a rich diversity of sand fly species and proven vectors [6]. In Belize to date, 8 of the 26 recorded sand fly species are indicated as known or suspected vectors [7–15] supporting infections with *L. (L)* *mexicana* [1, 10, 16], or *L. (Viannia)* *braziliensis* [17, 18], which cause cutaneous leishmaniasis (CL).

Early descriptions of CL from 1959, locally termed “bay sore” or “Chiclero’s ear”, were typical of *L*. (*L*.)* mexicana* infection [19], whereas later reports (1965–1970) described cases less characteristic of this species [14, 20]. When parasite identification became possible in 1984, infections among British troops training in Belize revealed *L. (L.) mexicana* in 9%, *L. (V.) braziliensis* in 86%, and unidentified species in 5% of cases [21]. Subsequent records show shifts in relative prevalence, but both species remain the major aetiological agents of CL in Belize [22–25]. Despite this well-documented history, systematic entomological surveillance in Belize has been limited. With anthropogenic changes in habitat cover and climate change, the lack of recent data constrains understanding of current transmission dynamics and hinders cross-border vector control coordination with Guatemala and Mexico, where CL is also endemic [7, 26].

Sand fly vectors in Belize typically inhabit forested environments, resting in leaf litter, tree hollows, and animal burrows [14]. Seasonal behaviour and species compositions are incompletely understood, though activity often peaks in the wet season [14], with some species more tolerant of drier conditions [27]. Extensive deforestation and land conversion continues to replace much of Belize’s primary forest with secondary or “mature” forest [28, 29] rendering many of the sand fly species’ historical location records obsolete.

These habitats, particularly in parts of north-west and central-east Belize, contain British Army’s Jungle Training Areas (JTAs), managed by the British Army Training Support Unit Belize (BATSUB) [25]. Each year, approximately 1,000 troops undergo temporary training in these areas, historically coinciding with subsequent clinical diagnosis of CL on return to the UK [22–25, 30, 31]. A recent 5.3-fold increase in CL annual cumulative incidence among British troops [25] further warrants the re-evaluation of sand fly species distributions in the region.

With access to these JTAs, this study aimed to characterise the spatiotemporal distribution, abundance, and species diversity of phlebotomine sand flies and *Leishmania* spp. in ecological zones in these regions of Belize, and to identify relative levels of risk of human exposure to sand flies leading to CL.

## Methods

### Study region and sampling sites

**Fig. 1:**
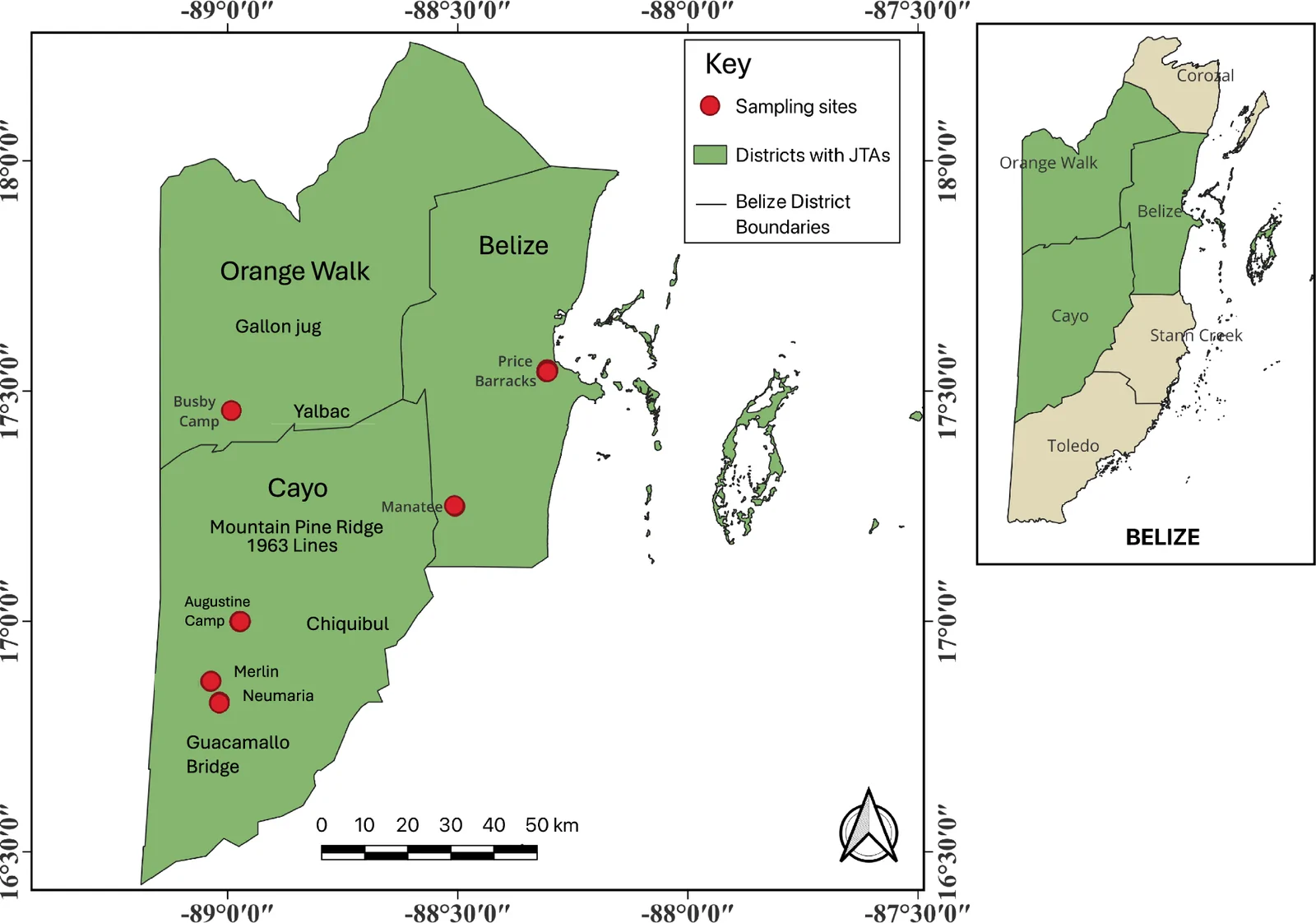
Location of study districts and sampling sites in Belize. The right-hand map shows Belize and the sampled districts shaded in green. The left-hand map shows the specific sampling site locations with these districts. Map created by the authors in QGIS v3.36 [32] using OpenStreetMap data (OpenStreetMap contributors) [33]

The study was conducted over a two-year period, from April 2023 to May 2025, sampling sand flies in six sentinel sites within JTAs in north-west and central-east Belize. Sites were geographically located in the districts of Cayo (three sites), Orange Walk (one site) and Belize (two sites) (Fig. 1). JTAs were purposefully chosen as CL case incidence data amongst the troops training in a specific JTA were systematically recorded, and could therefore be used to reliably estimate an area-specific Composite Exposure Risk Index (CERI) as detailed herein. JTAs are large areas (range 65–800 km^2^) with fixed sites where troops sleep and/or are nocturnally active over six weeks, after which they return to the UK.

#### Habitat descriptions

Vegetation cover in Cayo sampling sites included Caribbean pine systems in the Mountain Pine Ridge landscape alongside extensive evergreen broadleaf (hardwood) forest within the Chiquibul forest region [34]. In Orange Walk, the Busby Camp areas sit within a northern lowland mosaic that includes broadleaf hardwood and secondary palm forest, with pockets of pine forest and seasonally wet habitats such as marsh forest and freshwater lagoon margins [34, 35]. In Belize District, the JTAs are on the coastal plain with hardwood forest that also have extensive evergreen broadleaf. Across districts, patches of pine savanna habitats occur, and typically comprised mature pine with palmetto palms and a ground layer dominated by herbaceous flowering plants (notably grasses and sedges), scattered shrubs and small trees. The sample sites are further characterised in Table 1.

**Table 1:** Environmental characteristics of the JTA sampling sites

District	Sampling site	General characteristics [34, 35,36 ]	Altitude (m)	Climate^a^ [37]	
				Temperature °C (min–max)	Rainfall mm (min–max)
Belize	Price barracks	Peri-urban site (cement-wall buildings) with nearby secondary forest, where mangrove communities are prominent both along the mainland coast and in the Cayes (islands) (≈ 300 m due N and 30 m due SW)	12	23.9–31.5	200–400
Belize	Manatee	Secondary forest/jungle with mixed low, medium and high bush, where mangrove communities are prominent, and patches of pine savanna habitats with palmetto palms, herbaceous flowering plants, scattered shrubs and small trees	38		
Orange walk	Busby camp	Northern lowland mosaic that includes broadleaf hardwood and secondary palm forest, with pockets of pine forest and seasonally wet habitats such as marsh forest and freshwater lagoon margins	149	22.9–33.2	100–200
Cayo	Augustine camp	Upland Cayo setting associated with the Mountain Pine Ridge landscape: Caribbean pine-associated systems with adjacent secondary broadleaf (hardwood/palm) vegetation	452	23.3–32.4	150–300
Cayo	Merlin	Pine savanna–secondary forest mosaic: pine with palmetto palms and herbaceous flowering ground layer, adjacent to secondary broadleaf jungle	485		
Cayo	Neumaria	Broadleaf secondary jungle in the Chiquibul landscape, mainly medium and high bush (evergreen hardwood forest structure)	600		

^a^Humidity remains around 80% throughout the year, with a < 5% decrease during the dry season. Climate data were obtained from automatic weather stations (AWS) operated by the National Meteorological Service of Belize [38]

### Climate

Belize experiences two distinct climatic seasons: a dry season from December to April, and a wet season from May to November, with average annual temperatures ranging from 23 to 27 °C. During the wet season, the southern part of the country receives between 150 and 400 mm of rainfall monthly, while Orange Walk, Cayo and Belize Districts receive < 100 mm per month [39, 40 ]. Humidity is close to 80% year-round, dipping slightly during the dry-season months [37].

### CL incidence data

Military medical records from the Defence Medical Information Capability Programme (DMICP: [MOD restricted data]) were searched to identify confirmed cases of CL among personnel who had trained in the sampled JTAs from January 2019 to December 2025 [25]. Visiting troops undergo six weeks training in Belize JTAs soon after which they return to the UK. Only clinical cases confirmed by laboratory PCR or histopathology testing recorded by military medical practitioners were included in the current analyses. Case records were linked to specific JTAs based on documented deployment and training histories held by BATSUB. Only post-deployment cases were considered to ensure probable exposure within Belize.

### Sand fly sampling

**Fig. 2:**
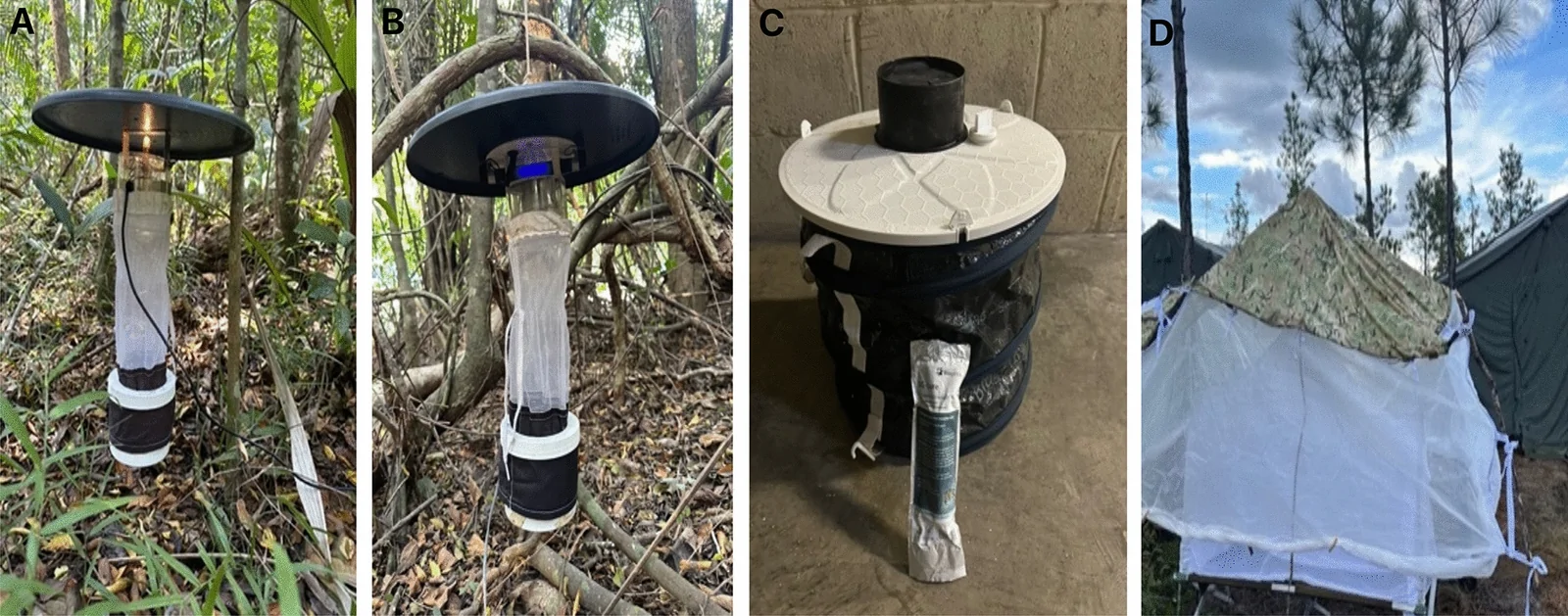
Traps used for sand fly sampling: **A** Centers for Disease Control and Prevention (CDC) light trap Model 512 (incandescent light bulb); **B** CDC trap (UV bulb); **C** BG-Sentinel traps and lure; **D** human landing catch (HLC) tent trap

Sand fly trapping was conducted during the dry seasons of 2024 and 2025 (February to March, and March to April, respectively), and the wet seasons of 2023 and 2024 (October to November). Traps were set on four consecutive nights at fixed locations in each of the six sampling sites in all seasons/years, except dry season 2025 when traps were set on two consecutive nights in all sites due to logistical constraints during the final sampling period, including limited field time and access to sampling sites. In each location, alternated trap types were arranged along a singe linear transect and spaced 30 m apart to minimise trap interference and to ensure spatial coverage of the sampling area. The entomological traps comprised 10 CDC light traps, six fitted with incandescent bulbs, and four fitted with ultra-violet (UV) bulbs (John W. Hock, Gainesville, Florida, USA), and four BG-Sentinel traps fitted with BG Lure attractant (Biogents, Regensburg, Germany) (Fig. 2A–C). All traps were operated between 18:00 and 06:00 h; BG-Sentinel traps were placed on the ground, and CDC light traps were suspended 30 cm above ground level. Trap positions were maintained consistently across sampling nights within each season to ensure comparability of collections.

In addition, human landing catches (HLC) were performed on four occasions in the six sampling sites on a single night each (total 24 trap nights), specifically located where training soldiers would routinely be camped at night. Tent traps (2 m × 0.7 m × 1 m; L × W × H) made of ultrafine woven mesh netting (0.28 mm × 0.79 mm) were erected over a smaller inner protective sand fly-proof net (4 m × 2 m × 2 m; L × W × H), in which an adult military volunteer remained between 17:00 and 20:00 h, provided with a cot bed (Fig. 2D). The volunteer inside the inner protective net did not use any torch or other light source during the trapping period. For the three hour trapping period, the lower edges of the outer net were raised 30 cm above the ground to allow sand fly access. At the end of the trapping period, the collector entered the outer net, lowered the net sides, and collected the sand flies inside the net using a torch and mechanical aspirator. It is assumed that sand flies entering the tent trap were attracted primarily to the human bait and would have landed on the volunteer, representing human-baited catches comparable to HLCs if the volunteer had not been protected by the inner net.

### Sand fly species identification

The collected sand flies were preserved in 70% ethanol and mounted on slides using CMCP-10 high-viscosity mounting fluid (Polysciences, Warrington, USA). Female specimens were prepared by separating the head and the last three abdominal segments, which contain the spermatheca, to support morphological identification, while retaining the remaining body parts for future investigations of natural infection. Male specimens were mounted whole. Morphological identification was carried out using the taxonomic keys of Galati, Ibáñez-Bernal and Young & Duncan [41–43]. Hereafter, sand fly species nomenclature followed Galati [41], and abbreviations for genera/subgenera follow Galati and Marcondes [44, 45].

To support interpretation of the epidemiological relevance of the fauna recorded, sand fly species were further classified as known or suspected *Leishmania* vectors in Belize based on published evidence from Belize and the wider Central American literature [1, 7, 10, 16–18, 46–53]. Confirmed vectors were defined as species with prior evidence of natural infection with *Leishmania* parasites and established epidemiological association with human transmission, and which are widely accepted as vector species in the Americas. Suspected vectors were defined as species for which flagellates or *Leishmania* spp. infection have been reported in field-collected specimens in Belize or elsewhere [14, 47, 51, 52, 54–58], but for which evidence of vector competence or epidemiological association remains incomplete. In the species inventory (Table S6), confirmed and suspected vectors are indicated by different superscripts.

### Measures of species relative abundance, diversity, richness and evenness

The total number of trap-nights, representing the sampling effort, was calculated as the product of the number of traps deployed and the number of nights operated at each site. Trapping success was expressed as the mean number of sand flies collected per trap per night, obtained by dividing the total number of sand flies captured at a site by the corresponding number of trap-nights.

#### Relative abundance (RA)

Relative abundance (RA) was calculated for each species as the proportion of individuals of that species out of the total number of sand flies captured at each site. This was calculated in Stata version 18.0 using the formula:

$$R{A}_{i}=\frac{{n}_{i}}{N},$$

where *n*_*i*_ is the number of individuals belonging to species *i* and *N* is the total number of individuals captured at the site. RA values range from low (0) to high (1 and were used to describe the composition and frequency of species across sites [59].

#### Species richness

Species richness was estimated using two non-parametric abundance-based estimators: the abundance-based coverage estimator (ACE) and Chao1. ACE estimates richness using the frequency structure of rare species, defined here as species represented by ≤ 10 individuals, and abundant species, defined as species represented by > 10 individuals. Chao1 estimates species richness using the number of singleton and doubleton species [60, 61].

Species accumulation curves were generated to assess sampling completeness across sites and seasons [62], and richness estimates were compared using ACE and Chao1 [60, 61].

The ACE estimator was calculated as:

$${\widehat{S}}_{ACE}={S}_{abund}+\frac{{S}_{rare}}{{C}_{ACE}}+\left(\frac{{F}_{1}}{{C}_{ACE}}\right){\gamma}_{ACE}^{2},$$

where *Ŝ*_ACE_ is estimated species richness, *S*_abund_ is the number of abundant species (typically with > 10 individuals), *S*_rare_ is the number of rare species (with ≤ 10 individuals), *F*_1_ is the number of species singletons among rare species, *C*_ACE_ is the sample coverage estimator for rare species and γ^2^_ACE_ is the estimated squared coefficient of variation for rare species frequencies [63]. Sample coverage for rare species was calculated as:

$${C}_{ACE}=1-\frac{{F}_{1}}{{N}_{rare}},$$

where *N*_rare_ is the total number of individuals from rare species.

The squared coefficient of variation for rare species frequencies was calculated from the frequency counts of rare species (*F*_i_, the number of species observed exactly i times for *i* = 1, …, 10) as:

$${\gamma}_{ACE}^{2}=max\left(0,\frac{{S}_{rare}{\sum}_{i=1}^{10}.i\left(i-1\right){F}_{i}}{{\left({\sum}_{i=1}^{10}.i{F}_{i}\right)}^{2}}- 1\right),$$

where *F*_i_ is the number of rare species observed exactly *i* times.

The Chao 1 estimator was calculated as:

$${\widehat{S}}_{Chao1}={S}_{obs}+\frac{{F}_{1}^{2}}{2{F}_{2}}$$, when *F*_2_ > 0.

When no doubletons were observed (*F*_2_ = 0), a bias-corrected lower-bound form was used to avoid division by zero:

$${\widehat{S}}_{Chao1}={S}_{obs}+\frac{{F}_{1\left({F}_{1}-1\right)}}{2\left({F}_{2}+1\right)},$$

where *S*_obs_ is the observed number species, *F*_1_ is the number of singleton species, and *F*_2_ is the number of doubleton species [64, 65].

#### Species diversity

Species diversity was assessed using Shannon’s Diversity Index (*H′*) and Pielou’s Evenness Index (*J′*). These indices were calculated in Stata. Shannon’s Diversity Index calculated as:

$${H}^{^{\prime}}=-\sum { p}_{i}ln\left({p}_{i}\right),$$

where *pi* is the proportion of each species [59, 66] (the relative abundance (RA) of species *i* in the sample, as calculated above).

Evenness was calculated using Pielou’s Evenness Index:

$${J}{\prime}=\frac{H}{ln\left(Sobs\right)},$$

where *H′* is Shannon’s Diversity Index and Sobs is the observed number of species in the sample [59, 67].

#### Species classifications

Species were classified according to their frequency of occurrence across catches as constant, accessory, or accidental. Constant species were present in more than 50% of catches, accessory species were present in 25–50% of catches, and accidental species were present in less than 25% of catches [68].

Dominance was classified using RA. Species with *RAᵢ* > 0.10 were classified as super-dominant, species with *RAᵢ* ≥ 0.05 to ≤ 0.10 as dominant, and species with *RAᵢ* < 0.05 as non-dominant. These thresholds were operationally defined for interpretation in the present study.

Species were also grouped by their RA based on the number of individuals captured: super-abundant, *ni* > 1000; very abundant, *ni* ≥ 500 to ≤ 1000; common, *ni* ≥ 100 to < 500; scattered, *ni* ≥ 50 to < 100; and rare, *ni* < 50. These categories were used descriptively to summarise species abundance patterns across the study sites.

#### Composite Exposure Risk Index (CERI)

A Composite Exposure Risk Index (CERI) was developed to estimate relative human exposure to *Leishmania* vectors across study sites and seasons. The index was designed following the OECD/JRC framework for composite indicator development and established approaches used in spatial vector-borne disease risk assessment [69–72]; calculations were performed in Stata. The index integrates three components: (1) the proportion of known *Leishmania* vector species among all sand fly species recorded per site (P); (2) RA of female sand flies (F); and (3) annual CL case incidence [25] (C)—the number of confirmed CL cases in the corresponding region, representing historical epidemiological evidence.

Each component was normalised to a 0–1 scale using min–max scaling to ensure comparability across differing units [72, 73]: ${X}_{i}{\prime}=\frac{{X}_{i}-{X}_{min}}{{X}_{max}-{X}_{min}},$ where *X*_*i*_ is the raw value of indicator *X* for unit _*i*_, *X*_*min*_ and *X*_*max*_ are the minimum and maximum values of *X* across all units included in the normalisation, and *X'*_*i*_ is the resulting min–max normalised value on the 0–1 scale. The composite score was calculated as the arithmetic mean of the three normalised components: $CERI=\frac{{P}{\prime}+{F}{\prime}+{C}{\prime}}{3}.$

Equal weighting was applied in accordance with OECD guidance when no empirical basis exists for differential weighting. Continuous CERI values (0–1) were categorised into three exposure strata for interpretability: low risk (< 0.10), medium risk (0.10–0.30), and high risk (> 0.30), aligning with commonly used risk-prediction benchmarks [74].

To reduce bias from zero-inflated case counts, a log-adjusted version of the third component was also computed: ${C}_{log}{\prime}=ln\left(Cases+0.5\right)$ and re-normalised to the same 0–1 scale before recombining a $CER{I}_{log}=\frac{{P}{\prime}+{F}{\prime}+{C}_{log}{\prime}}{3}$. This adjustment prevented zero-case collapse and allowed approximate 95% confidence intervals to be derived for the log-transformed component. Comparison of CERI and CERIₗₒg confirmed that the final risk rankings were unchanged (i.e. no meaningful differences in the overall outcome), with only negligible category reassignments that did not affect the overall interpretation. To stabilise proportion estimates for sites with very small sample sizes (n ≤ 10), the Jeffreys correction $\left(Beta\left[\mathrm{0.5,0.5}\right]prior\right)$ was applied to the proportion of vector species $\left[P=\frac{k+0.5}{n+1}\right]$ following Brown et al. [75] and Jeffreys [76]. Categorised risk levels were visualised as a season-by-site heatmap (Fig. 11).

### Statistical analyses

Descriptive statistics were used to summarise trap counts, sex ratios, and site-level differences in sand fly captures across seasons and years. Sand fly trapping success data were log_10_-transformed $\left[log\left(mean value+0.01\right)\right]$ to reduce the influence of zero-heavy and right-skewed data distribution. Sex-specific trapping success was compared using paired t-tests to assess mean differences in male and female counts. Differences in sex ratios between wet and dry seasons were tested using Pearson’s Chi-square test (χ^2^). Site-level variation in total trapping success was examined using z-tests for proportions, comparing each site’s contribution to the total catch against the overall mean proportion of captures.

To examine temporal and spatial variation in abundance and diversity while accounting for repeated sampling at the same sites (autocorrelation), multivariate analyses, including linear and generalised linear mixed-effects models (LMMs/GLMMs), were fitted using the *lme4* package in R. Log_10_-transformed trapping success data were modelled using a Gaussian LMM, and species richness (number of sand fly species per site-season) using a Poisson GLMM. Both models included *season* (wet/dry), *year* (Year 1/Year 2), and their interaction as fixed effects. Sampling site was included as a random effect.

Model assumptions were checked by inspecting residual plots and normality of random effects. Effect sizes (β coefficients ± SE) were reported with corresponding *z* or *t* statistics and *p*-values. For the LMM, between-site variance and intraclass correlation coefficients (ICC) were estimated to quantify the proportion of variance explained by site-level clustering.

## Results

### Sampling effort and species collected

A total of 7,759 phlebotomine sand fly specimens belonging to 20 species in 10 genera (Table 3) were collected in 936 trap nights over two years of standardised trapping. Of these, 4,032 were trapped during the wet seasons (52%: 2,171 in Year 1; 1,861 in Year 2), and 3,727 during the dry seasons (48%: 3,098 in Year 1; 629 in Year 2) (Table 2). The overall female-to-male ratio was 2.1:1 (5,248 females; 2,511 males). Detailed analyses of sex ratios by season, site, and species are presented in the Supplementary Material (Table S1).

**Table 2:** Sand fly counts, trap nights, and trapping success by site and annual season

Year	Season	Measure	Price barracks	Manatee	Busby camp	Augustine camp	Merlin	Neumaria	HLC	Total specimens
1	Wet season	Sand fly count	0	5	280	226	232	1400	28	2171
		Trap nights	112	56	14	56	56	56	12	362
		Trapping success	0.00	0.09	20.00	4.04	4.14	25.00	2.3	6
	Dry season	Sand fly count	1	630	133	221	319	1741	53	3098
		Trap nights	56	28	28	28	28	28	6	202
		Trapping success	0.02	22.50	4.75	7.89	11.39	62.18	8.8	15.3
2	Wet season	Sand fly count	0	108	225	182	195	1137	14	1861
		Trap nights	56	28	28	28	28	28	6	202
		Trapping success	0.00	3.86	8.04	6.50	6.96	40.61	2.3	9.2
	Dry season	Sand fly count	0	2	47	71	42	467	0	629
		Trap nights	28	28	28	28	28	28	2	170
		Trapping success	0.00	0.07	1.68	2.54	1.50	16.68	0	3.7
Total specimen			1	745	685	700	788	4745	95	7759

*HLC* human landing catches

**Fig. 3:**
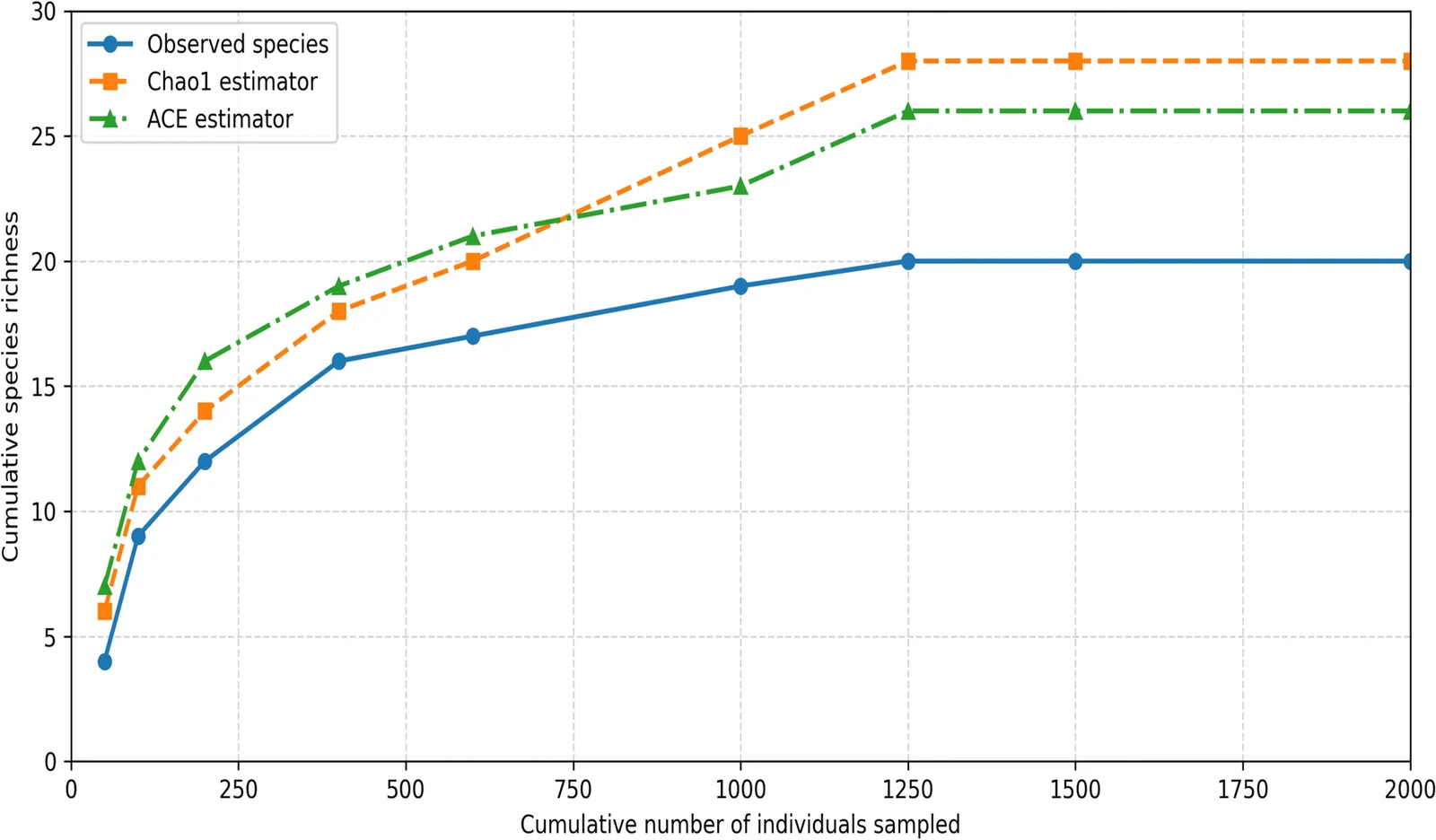
Cumulative species richness and estimators (ACE, Choa1) of the number of sand fly species in the study region. The x-axis is truncated at 2,000 individuals to provide a clearer view of the early accumulation pattern. The curve had already reached a plateau (20 species) well before the total sample size of 7,759 sand flies collected

The cumulative number of species identified (species richness) rose rapidly during early sampling, and reached a plateau at 20 species after capturing approximately 1,250 specimens, the equivalent of 16% of the cumulative trap nights. The ACE and Chao1 estimators predicted 26.2 and 28 species, respectively (approximately six to eight species more than observed) (Fig. 3), indicating a reasonably good sampling coverage, though a number of species in the study region may not have been detected. At least seven species previously recorded from across Belize were not collected in the present study (Table S6).

The observed sand fly assemblage was dominated by *Da. deleoni* (37.6%), *Ty. triramula* (24.2%), *Pi. ovallesi* (11.4%), *Br. mesai* (8.9%), and *Ps. panamensis* (8.8%), which together comprised more than 90% of captures. *Dampfomyia*, *Psathyromyia* and *Psychodopygus* were the most diverse genera, with three species each. Several species, including *Ps. geniculatus*, *Pa. undulata* and *Lu. longipalpis*, were rare or accidental.

Trapping included human landing catches (HLCs) which yielded 95 specimens (81 females and 14 males), representing eight species, *Ps. panamensis* (23.5%), *Pi. ovallesi* (21%), *Ty. triramula* (21%), *Da. deleoni* (17.3%), *Ny. ylephiletor* (8.6%), *Lu. cruciata* (3.7%), *Bi. olmeca olmeca* (3.7%), and *Ps. bispinosus* (1.2%).

### Vector compositions

**Fig. 4:**
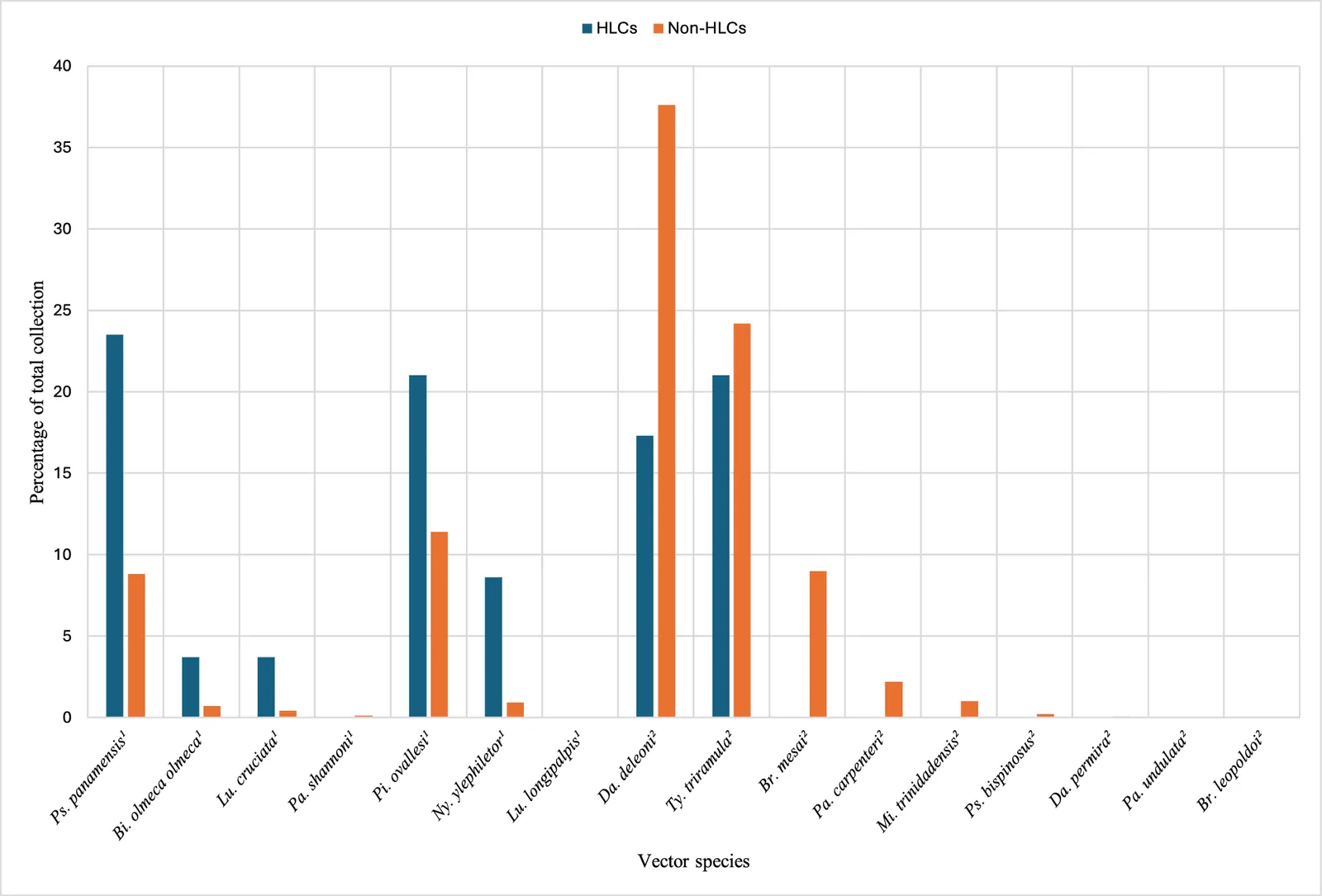
Comparison of known vector species compositions between human landing catches (HLCs) and non-human landing catches (Non-HLCs) traps. ^1^ Known vector species, ^2^ suspected vector species

Seven (35%) of the 20 species detected in this study are known vectors of *Leishmania* spp. parasites, namely *Ps. panamensis, Pi. ovallesi, Bi. olmeca olmeca, Ny. ylephiletor, Lu. cruciata, Pa. shannoni* and *Lu. longipalpis*. A further 9 of the 20 species are suspected vectors (Table 3). Confirmed vector species dominated collections, both amongst those captured by HLCs (60.5% of all captures), and by non-HLC methods (22.3%). Predominant vector species captured by HLCs were five compared to seven in non-HLCs (Fig. 4).

**Table 3:** Estimates of sand fly relative abundance, consistency and dominance in sampling areas

*Species*	Total	Ratio ♀/♂	RA	Rank	Dominance	Abundance	Species consistency
*Dampfomyia deleoni*^b^	2883	8.74	0.3762	1	SD	SA	Constant
*Trichopygomyia triramula*^b^	1851	1.34	0.2415	2	SD	SA	Constant
*Pintomyia ovallesi*^a^	876	0.69	0.1143	3	SD	VA	Constant
*Brumptomyia mesai*^b^	686	0.66	0.0895	4	D	VA	Constant
*Psychodopygus panamensis*^a^	672	2.69	0.0877	5	D	VA	Constant
*Psathyromyia carpenteri*^b^	170	0.73	0.0222	6	ND	C	Accessory
*Micropygomyia trinidadensis*^b^	79	0.70	0.0103	7	ND	S	Accessory
*Nyssomyia ylephiletor*^a^	67	0.81	0.0087	8	ND	S	Accessory
*Bichromomyia olmeca olmeca*^a^	51	0.38	0.0067	9	ND	S	Accessory
*Lutzomyia cruciata*^a^	29	0.81	0.0038	10	ND	R	Accidental
*Psychodopygus bispinosus*^b^	18	17.00	0.0023	11	ND	R	Accidental
*Pintomyia serrana*	17	0.06	0.0022	12	ND	R	Accidental
*Dampfomyia disneyi*	17	♂	0.0022	12	ND	R	Accidental
*Psathyromyia shannoni*^a^	11	0.22	0.0014	13	ND	R	Accidental
*Dampfomyia permira*^b^	3	1.00	0.0005	14	ND	R	Accidental
*Psychodopygus geniculatus*	2	♀	0.0003	15	ND	R	Accidental
*Psathyromyia undulata*^b^	1	♀	0.0001	16	ND	R	Accidental
*Lutzomyia longipalpis*^a^	1	♂	0.0001	16	ND	R	Accidental
*Brumptomyia leopoldoi*^b^	1	♂	0.0001	16	ND	R	Accidental
*Psathyromyia soccula*	1	♂	0.0001	16	ND	R	Accidental
Unknown Spp	228	14.00	–	–	–	–	–
Total	7664	2.1:1					

*RA* relative abundance. Dominance: *SD* super dominant, *D* dominant, *ND* non-dominant. Abundance: *SA* super abundant, *VA* very abundant, *C* common, *S* scattered, *R* rare

^a^Known sand fly vector species

^b^Suspected sand fly vector species, in Belize and other countries in the region. Unknown Spp are specimens that could not be reliably assigned to subtribe level because the diagnostic morphological characters required for confirmation were not sufficiently preserved or visible

### Trapping success across sites, seasons and years

**Fig. 5:**
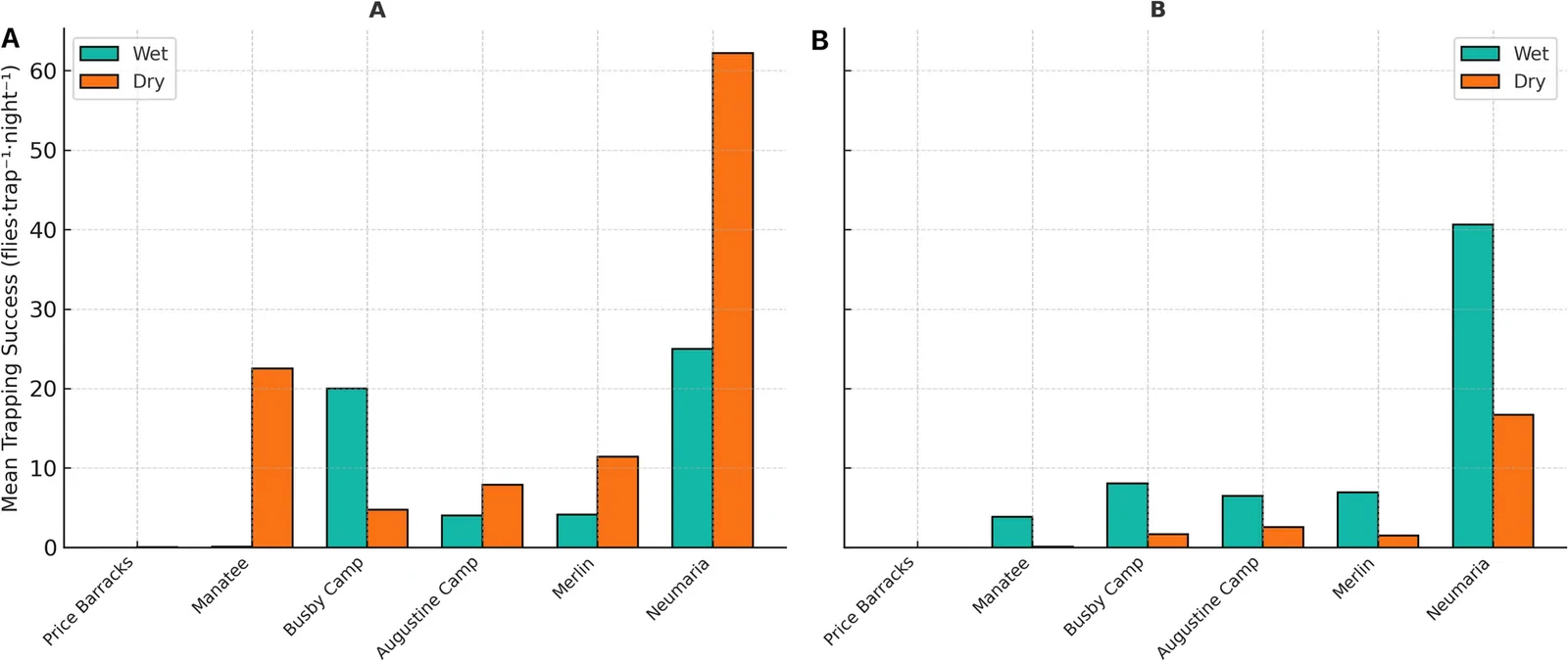
Mean trapping success in sampling sites in the wet and dry seasons of year 1 (**A**) and year 2 (**B**)

Mean trapping success was 8.2 flies trap⁻1 night⁻1, ranging from 3.7 (dry Season, Year 2) to 15.3 (dry Season, Year 1). Across sites, trapping success ranged from 0.00 to 62.2 flies trap⁻1 night⁻1, with highest numbers at Neumaria and lowest at Price Barracks (PB), where only a single sand fly was captured over the study period (Table 2; Fig. 5). Neumaria accounted for 61.9% of all captures (4,745 of 7,759 specimens), significantly higher than in other locations (Z =  − 2.95, *P* < 0.001).

Accounting for site-level clustering in overall trapping success (ICC = 0.740, 95% CI 0.375–0.931; Table S4), mean trapping success in the dry-season was ~ 72% lower in Year 2 than in Year 1 (*P* = 0.001). Wet-season catches tended to be marginally lower than dry-season catches in Year 1 (*P* = 0.065), whereas in Year 2, the wet-season catches were 2.5-fold higher than in the dry-season (test of season × Year interaction term, *P* = 0.002).

### Species spatial distributions

**Fig. 6:**
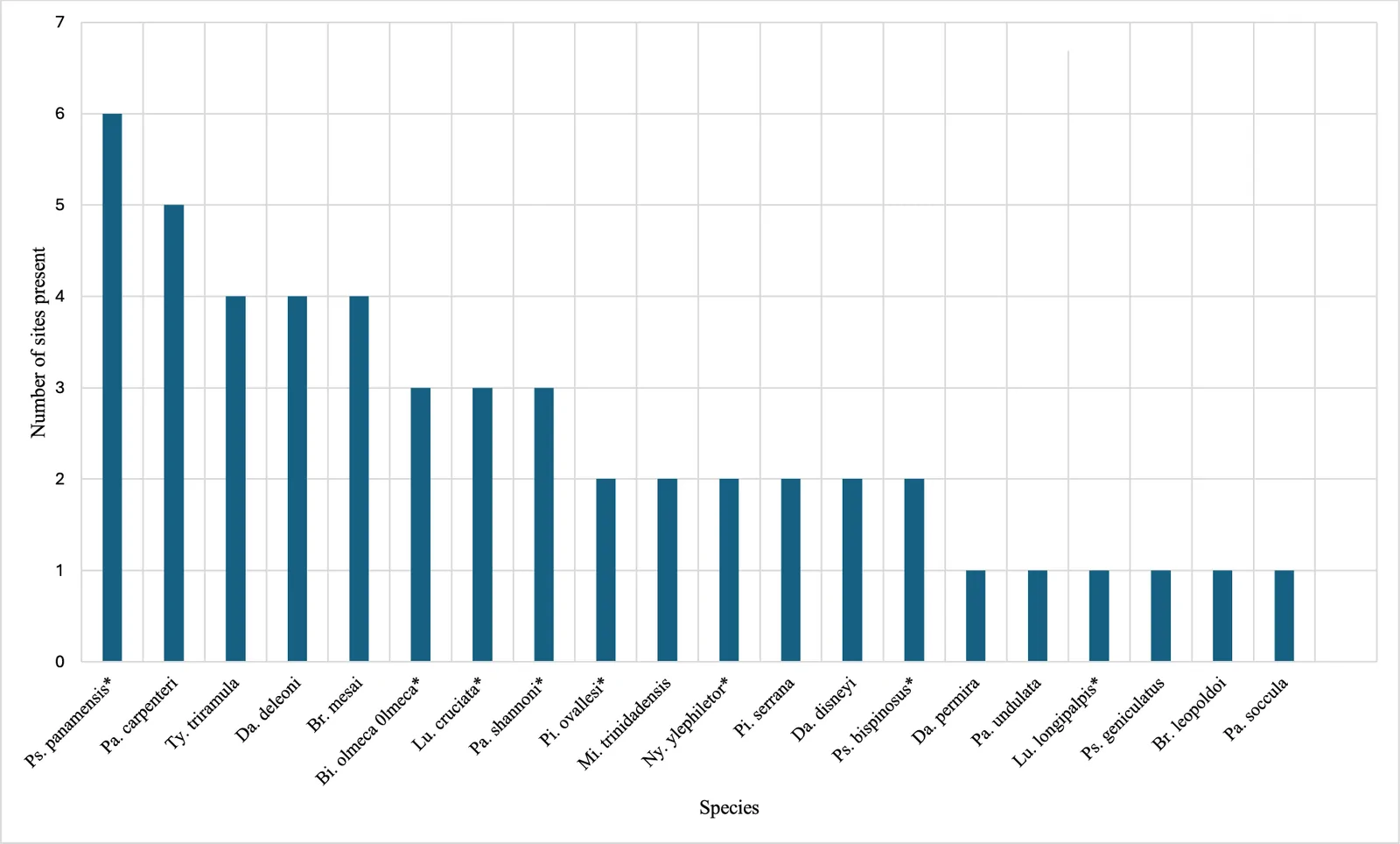
The number of sites of six sites sampled where sand fly species presence was recorded (ordered from highest to lowest)

**Fig. 7:**
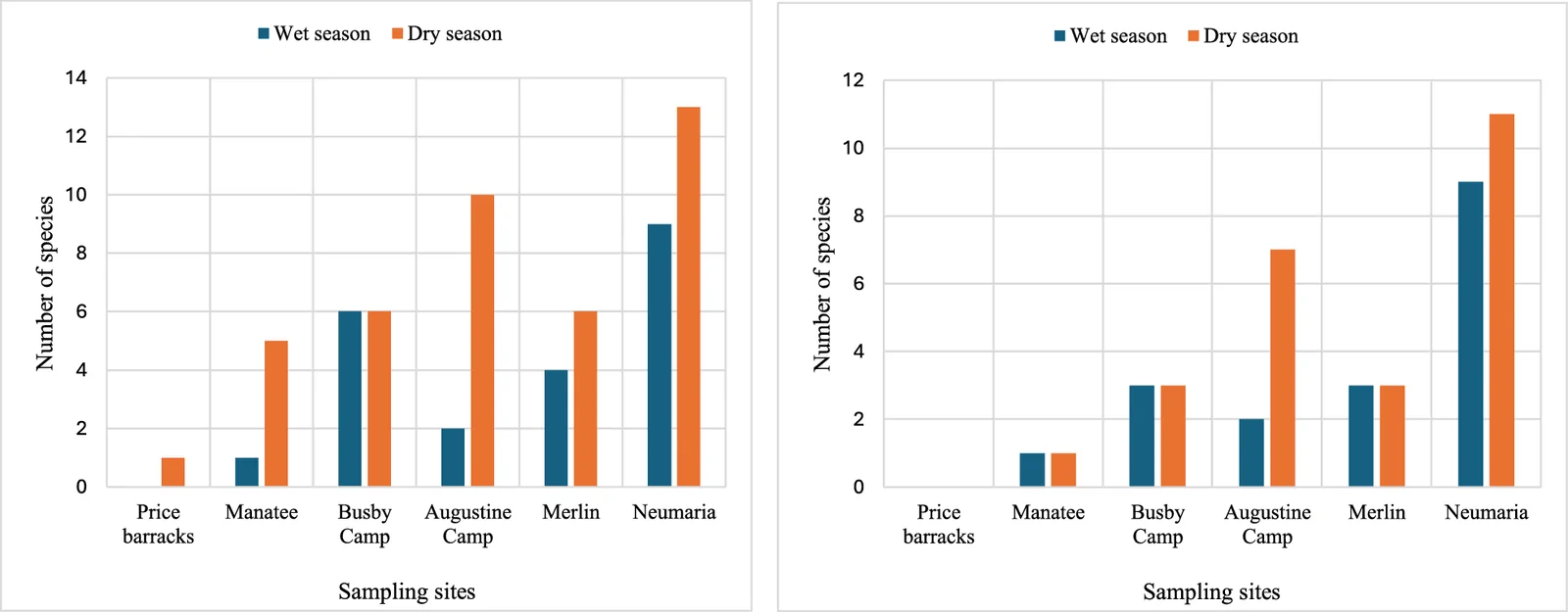
Species richness in the six sampling site by season in **A** year 1; **B** year 2. Refer to Tables 4, 5, 6, 7 for the associated species compositions

Sand fly species spatial occurrence varied markedly between sampling sites. *Ps. panamensis* and *Pa. carpenteri* were the most widespread, detected at ≥ 80% of sites. *Da. deleoni*, *Ty. triramula*, and *Br. mesai, Lu cruciata, Pa shannoni and Bi olmeca olmeca* occurred in ≥ 50% of locations, while all other species were more restricted to one or two sites (Fig. 6; Tables 4, 5, 6, 7). *Lu. longipalpis*, the main vector of *L. infantum*, and *Psathyromyia soccula*, appeared only at Merlin and Neumaria, respectively, marking their first detection in Belize. Price Barracks showed absence or near-absence of sand flies, while Manatee consistently recorded very low sand fly abundance across seasons (Fig. 7; Tables 4, 5, 6, 7).

**Table 4:** Distribution, abundance, and diversity of sand fly species across different sampling sites in the wet season (year 1)

Species	Price barracks			Manatee			Busby camp			Augustine camp			Merlin			Neumaria			Total specimen	% of total
	**♀**	♂	%	♀	♂	%	♀	♂	%	♀	♂	%	♀	♂	%	♀	♂	%		
*Dampfomyia deleoni*	–	–	–	–	–	–	5	6	3.9	–	–	–	–	–	–	1077	68	81.8	1156	53.9
*Trichopygomyia triramula*	–	–	–	4	1	100	4	0	1.4	134	63	87.2	103	79	78.5	–	–	–	388	18.1
*Psychodopygus panamensis*^a^	–	–	–	–	–	–	157	68	80.4	25	4	12.8	35	–	15.1	31	7	2.7	327	15.3
*Brumptomyia mesai*	–	–	–	–	–	–	3	6	3.2	–	–	–	–	–	–	42	65	7.6	116	5.4
*Pintomyia ovallesi*^a^	–	–	–	–	–	–	–	–	–	–	–	–	–	–	–	61	–	4.4	61	2.9
*Bichromomyia olmeca olmeca*^a^	–	–	–	–	–	–	–	–	–	–	–	–	6	4	4.3	5	3	0.6	18	0.8
*Psathyromyia carpenteri*	–	–	–	–	–	–	–	18	6.4	–	–	–	–	–	–	–	–	–	18	0.8
*Psychodopygus bispinosus*^a^	–	–	–	–	–	–	–	–	–	–	–	–	5	–	2.2	10	–	0.7	15	0.7
*Nyssomyia ylephiletor*^a^	–	–	–	–	–	–	–	–	–	–	–	–	–	–	–	4	–	0.3	4	0.2
*Micropygomyia trinidadensis*	–	–	–	–	–	–	–	–	–	–	–	–	–	–	–	6	–	0.4	6	0.3
*Psathyromyia shannoni*^a^	–	–	–	–	–	–	–	4	1.4	–	–	–	–	–	–	–	–	–	4	0.2
*Brumptomyia leopoldoi*	–	–	–	–	–	–	–	–	–	–	–	–	–	–	–	–	1	0.1	6	0.3
Unknown spp^β^	–	–	–	–	–	–	9	–	3.2	–	–	–	–	–	–	20	–	1.5	30	1.4
Total specimen	–	–		4	1		178	102		159	67		149	83		1256	144		2143	100
Species richness	0			1			6			2			4			9			12	
Shannon Diversity Index (H′)	0			0			0.702			0.383			0.694			0.701				
Evenness (J′)	0			0			0.392			0.553			0.501			0.319				

^a^Sand fly species known to transmit leishmaniasis in Belize and other countries in the region

^β^Unidentified including gravid specimens

**Table 5:** Distribution, abundance, and diversity of sand fly species across different sampling sites in the dry season (year 1)

Species	Price barracks			Manatee			Busby camp			Augustine camp			Merlin			Neumaria			Total specimen	% of total
	♀	♂	%	♀	♂	%	♀	♂	%	♀	♂	%	♀	♂	%	♀	♂	%		
*Trichopygomyia triramula*	–	–	–	244	328	90.8	–	2	1.5	41	14	24.9	148	162	97.2	–	–	–	939	30.8
*Pintomyia ovallesi*^a^	–	–	–	–	–	–	–	–	–	–	–	–	1	–	0.3	149	466	35.3	616	20.2
*Dampfomyia deleoni*	–	–	–	2	2	0.6	97	5	76.7	121	9	58.8	–	–	–	206	107	18.0	549	18.0
*Brumptomyia mesai*	–	–	–	–	–	–	–	2	1.5	1	–	0.5	2	–	0.6	138	293	24.8	436	14.3
*Psathyromyia carpenteri*	–	–	–	1	–	0.2	8	3	8.3	1	2	1.4	–	1	0.3	54	65	6.8	135	4.4
*Psychodopygus panamensis*^a^	1	–	100	1	1	0.3	1	4	3.8	4	7	5.0	1	–	0.3	12	23	2.0	55	1.8
*Micropygomyia trinidadensis*	–	–	–	–	–	–	–	–	–	1	–	0.5	–	–	–	18	37	3.2	56	1.8
*Nyssomyia ylephiletor*^a^	–	–	–	–	–	–	–	–	–	1	–	0.5	–	–	–	11	29	2.3	41	1.4
*Lutzomyia cruciata*^a^	–	–	–	2	–	0.3	–	–	–	5	4	4.1	–	–	–	4	8	0.7	23	0.8
*Dampfomyia disneyi*	–	–	–	–	–	–	–	–	–	–	8	3.6	–	–	–	–	3	0.2	11	0.4
*Psathyromyia shannoni*^a^	–	–	–	–	–	–	1	1	1.5	–	–	–	1	–	0.3	–	4	0.2	7	0.2
*Dampfomyia permira*	–	–	–	–	–	–	–	–	–	–	–	–	–	–	–	1	2	0.2	3	0.1
*Psychodopygus bispinosus*^a^	–	–	–	–	–	–	–	–	–	–	–	–	–	–	–	1	1	0.1	2	0.1
*Psathyromyia undulata*	–	–	–	–	–	–	–	–	–	1	–	0.5	–	–	–	–	–	–	1	0.03
*Psathyromyia soccula*	–	–	–	–	–	–	–	–	–	–	–	–	–	–	–	–	1	0.1	1	0.03
Unknown spp^β^	–	–	–	48	1	7.8	9	–	6.8	1		0.5	3	–	0.9	97	11	6.20	170	5.6
Total specimen	1	0		298	332		116	17		177	44		156	163		691	1050		3045	100
Species richness	1			5			6			10			6			13			15	
Shannon Diversity Index (H′)	0			0.100			0.705			1.215			0.124			1.601				
Evenness (J′)	0			0.062			0.393			0.528			0.069			0.624				

^a^Sand fly species known to transmit leishmaniasis in Belize and other countries in the region

^β^Unidentified including gravid specimens

**Table 6:** Distribution, abundance, and diversity of sand fly species across different sampling sites in the wet season (year 2)

Species	Price barracks			Manatee			Busby camp			Augustine camp			Merlin			Neumaria			Total specimen	% of total
	♀	♂	%	♀	♂	%	♀	♂	%	♀	♂	%	♀	♂	%	♀	♂	%		
*Dampfomyia deleoni*	–	–	–	–	–	–	8	1	4.0	–	–	–	–	–	–	911	78	87.0	998	54.0
*Trichopygomyia triramula*	–	–	–	95	13	100	–	–	–	139	40	98.4	107	64	87.7	–	–	–	458	24.8
*Psychodopygus panamensis*^a^	–	–	–	–	–	–	171	41	94.2	3	–	1.7	17	–	8.7	28	12	3.5	272	14.7
*Brumptomyia mesai*	–	–	–	–	–	–	1	3	1.8	–	–	–	–	–	–	16	33	4.3	53	2.9
*Pintomyia ovallesi*^a^	–	–	–	–	–	–	–	–	–	–	–	–	5	–	2.6	23	3	2.3	31	1.7
*Bichromomyia olmeca olmeca*^a^	–	–	–	–	–	–	–	–	–	–	–	–	–	–	–	–	4	0.4	4	0.2
*Psychodopygus bispinosus*^a^	–	–	–	–	–	–	–	–	–	–	–	–	–	–	–	1	–	0.1	1	0.1
*Nyssomyia ylephiletor*^a^	–	–	–	–	–	–	–	–	–	–	–	–	–	–	–	11	–	1.0	11	0.6
*Micropygomyia trinidadensis*	–	–	–	–	–	–	–	–	–	–	–	–	–	–	–	3	–	0.3	3	0.2
*Dampfomyia disneyi*	–	–	–	–	–	–	–	–	–	–	–	–	–	–	–	–	5	0.4	5	0.3
Unknown spp^β^	–	–	–	–	–	–	–	–	–	–	–	–	2	–	1.0	9	–	0.8	11	0.6
Total specimen	–	–		95	13		180	45		142	40		131	64		1002	135		1847	100
Species richness	0			1			3			2			3			9			10	
Shannon Diversity Index (H′)	0			0			0.256			0.084			0.416			0.568				
Evenness (J′)	0			0			0.233			0.121			0.379			0.259				

^a^Sand fly species known to transmit leishmaniasis in Belize and other countries in the region

^β^Unidentified including gravid specimens

**Table 7:** Distribution, abundance, and diversity of sand fly species across different sampling sites in the dry season (year 2)

**Species**	Price barracks			Manatee			Busby camp			Augustine camp			Merlin			Neumaria			Total specimen	% of total
	♀	♂	%	♀	♂	%	♀	♂	%	♀	♂	%	♀	♂	%	♀	♂	%		
*Trichopygomyia triramula*	–	–	–	2	–	100	–	–	–	17	7	33.8	22	18	95.2	–	–	–	66	10.5
*Pintomyia ovallesi*^a^	–	–	–	–	–	–	–	–	–	–	–	–	–	–	–	120	48	36.0	168	26.7
*Dampfomyia deleoni*	–	–	–	–	–	–	30	9	83.0	29	4	46.5	–	–	–	101	7	23.1	180	28.6
*Brumptomyia mesai*	–	–	–	–	–	–	–	–	–	–	–	–	–	–	–	70	11	17.3	81	12.9
*Psathyromyia carpenteri*	–	–	–	–	–	–	2	–	4.3	–	–	–	–	–	–	6	9	3.2	17	2.7
*Psychodopygus panamensis*^a^	–	–	–	–	–	–	–	2	4.3	1	6	9.9	1	–	2.4	1	7	1.7	18	2.9
*Micropygomyia trinidadensis*	–	–	–	–	–	–	–	–	–	–	–	–	–	–	–	5	10	3.2	15	2.4
*Nyssomyia ylephiletor*^a^	–	–	–	–	–	–	–	–	–	–	–	–	–	–	–	3	8	2.4	11	1.8
*Bichromomyia olmeca olmeca*^a^	–	–	–	–	–	–	–	–	–	–	1	1.4	–	–	–	3	25	6.0	29	4.6
*Pintomyia serrana*	–	–	–	–	–	–	–	–	–	1	–	1.4	–	–	–	–	16	3.4	17	2.7
*Lutzomyia cruciata*^a^	–	–	–	–	–	–	–	–	–	2	2	5.6	–	–	–	–	2	0.4	6	1.0
*Dampfomyia disneyi*	–	–	–	–	–	–	–	–	–	–	1	1.4	–	–	–	–	–	–	1	0.2
*Psychodopygus geniculatus*	–	–	–	–	–	–	–	–	–	–	–	–	–	–	–	2	–	0.2	2	0.3
*Lutzomyia longipalpis*^a^	–	–	–	–	–	–	–	–	–	–	–	–	–	1	2.4	–	–	–	1	0.2
Unknown spp^β^	–	–	–	–	–	–	4	–	8.5	–	–	–	–	–	–	10	3	2.8	17	2.9
Total specimen	0	0		2	0		36	11		50	21		23	19		321	146		629	100
Species richness	0			1			3			7			3			11			14	
Shannon Diversity Index (H′)	0			0			0.374			1.179			0.431			1.741				
Evenness (J′)	0			0			0.340			0.732			0.268			0.726				

^a^Sand fly species known to transmit leishmaniasis in Belize and other countries in the region

^β^Unidentified including gravid specimens

### Species richness, diversity, and vector dominance

**Fig. 8:**
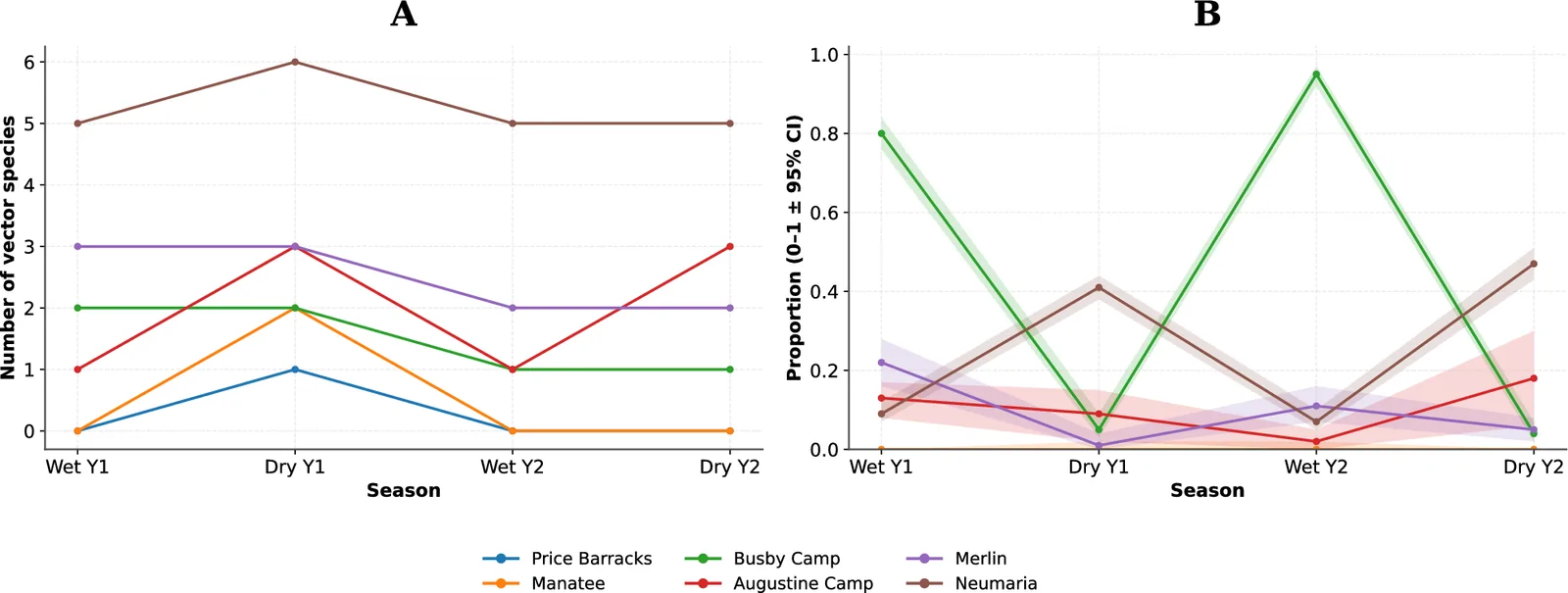
Variation in the number and proportion of sand flies that are known vectors across sampling sites and wet and dry seasons.** A** Number of vector species; **B** proportion of total species captured (shaded areas: 95% C.I.s)

The total number of sand fly species was greatest in the first dry season at Augustine Camp (*n* = 10 species) and Neumaria (*n* = 13), and lowest in PB and Manatee (Figs. 7, 8).

**Fig. 9:**
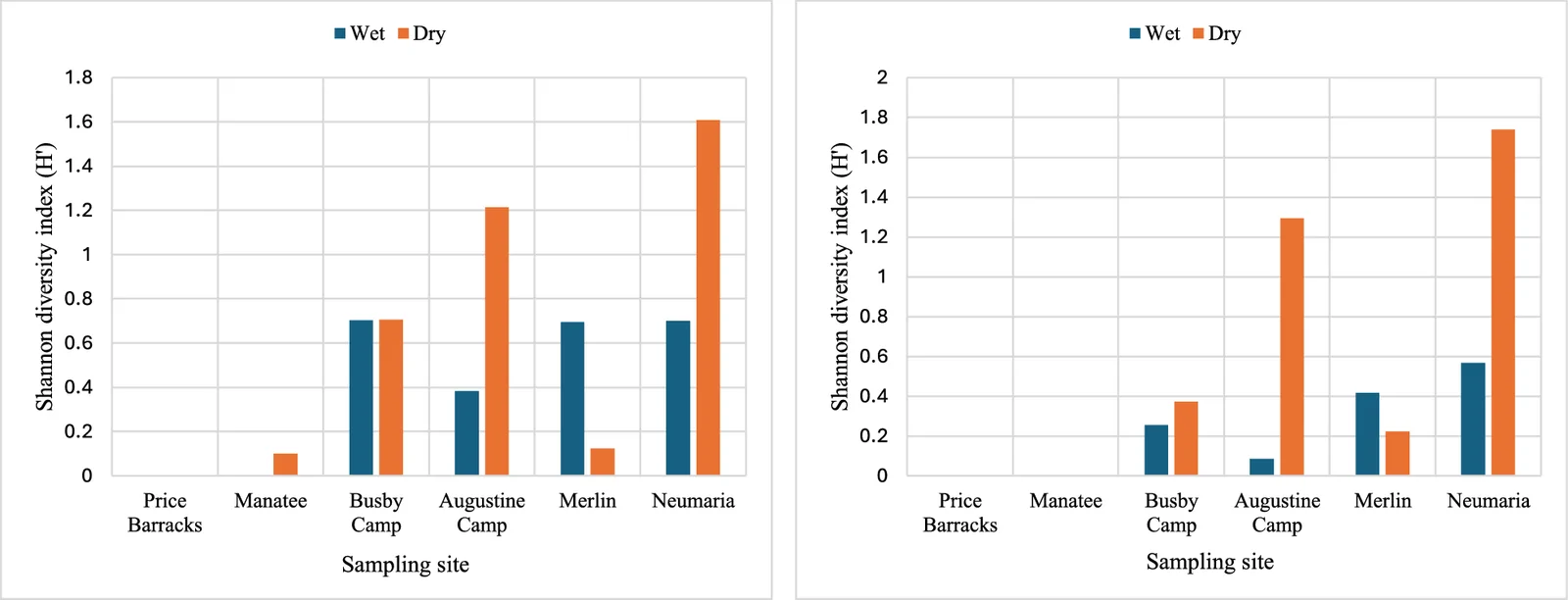
Shannon diversity (H′) of sand fly species across sites, season and year. **A**—year 1; **B—**year 2

**Fig. 10:**
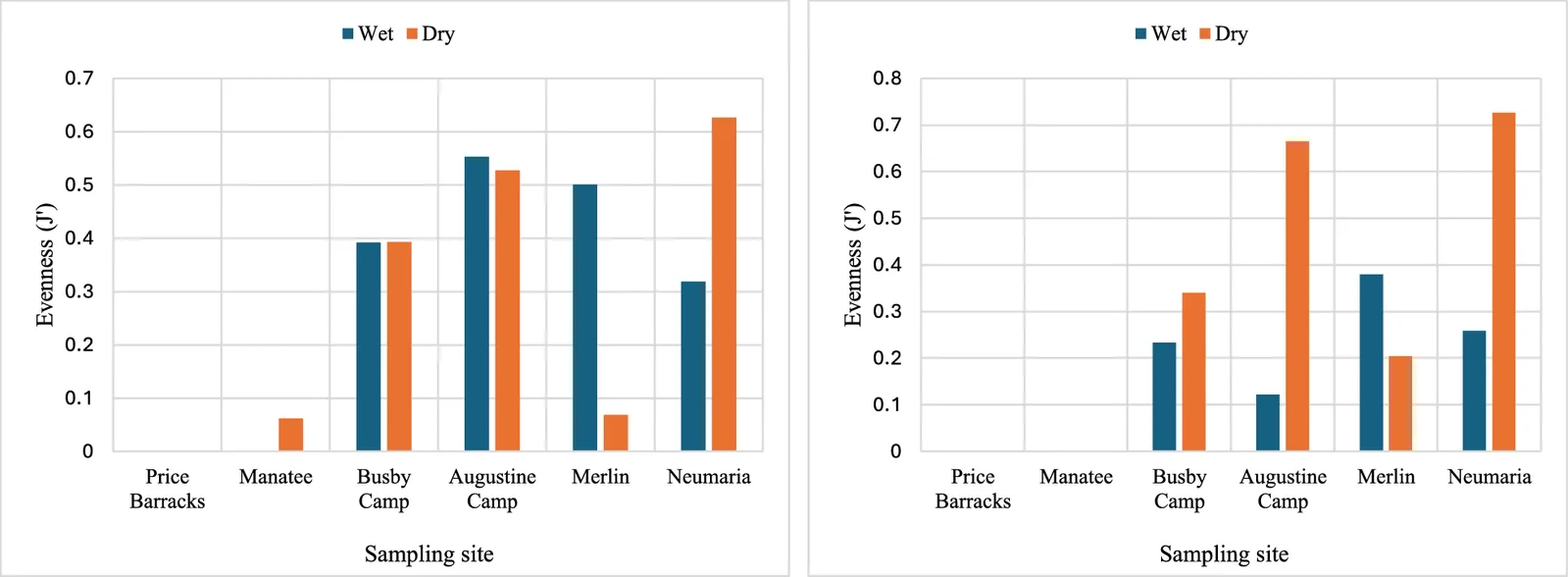
Evenness (J′) in sand fly species across sampling sites and season in year 1 (**A)** and year 2 (**B)**

Species diversity indices mirrored the site contrasts in trapping success. Neumaria also showed the highest Shannon diversity (H′ = 1.60–1.74) and evenness (J′ = 0.64–0.73), followed by Augustine Camp (H′ ≈ 1.17–1.22), whereas PB had the lowest diversity (H′ ≈ 0) (Figs. 9, 10; Tables 4, 5, 6, 7). Differences in species richness between Neumaria and all other five sites combined were significant in both seasons (*P* < 0.001).

Species richness tended to be lower in Year 2 than in Year 1 (*P* = 0.051), and was approximately 46% lower in the wet season compared to the dry season (*P* = 0.018). The season × year interaction term was not significant (*P* = 0.47). Substantial site-to-site variation remained (random-effect variance = 0.816), and the random site effect was strongly supported (LR test vs fixed-effects Poisson model, *P* < 0.001; Table S4).

The number of known vectors were highest in Neumaria (5–6 species per season), and lowest at coastal sites PB (one specimen of *Ps. panamensis*) and Manatee (two specimens each of *Ps. panamensis* and *Lu. cruciata*) (Fig. 8A), indicating minimal vector presence and limited suitability for sustained vector activity at the latter two sites. The proportion of total sand fly species classified as confirmed vectors by site and season (Table S3), indicated vector dominance to be highest across both year wet seasons at Busby Camp (80–94%) and where *Ps. panamensis* was the most abundant vector species, while at Neumaria vector dominance was highest in both dry seasons (41–47%) with *Pi. ovallesi* being the most abundant species. Augustine Camp and Merlin showed moderate proportions of vector species (5–22%) with minor seasonal fluctuation (Fig. 8B).

### Composite exposure risk index (CERI)

**Fig. 11:**
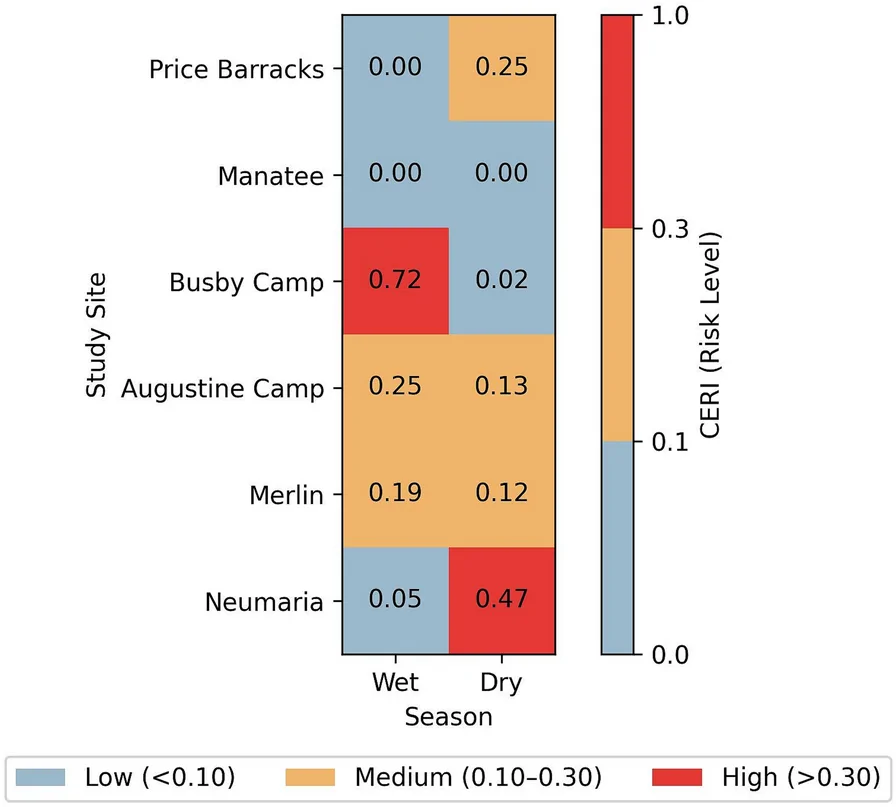
Classification of the Composite Exposure Risk Index (CERI) values calculated across sampling sites and seasons

Forty-nine confirmed clinical cases of CL was recorded amongst 2,100 military personnel deployed in the entomologically sampled JTAs from 2019 to 2025 (Table S5). During this interval, the CL 7-year period incidence ranged from 33 (33/950) in Busby Camp, to 0 (0/400) in Manatee (Table S5). Using these CL case data, trapping success of vector species, and number of vector species as proportions of total species captured per site, CERI values were calculated, representing an estimated risk of human-sand fly interaction leading to clinical CL. The highest CERI values were associated with Busby Camp during the wet season (CERI = 0.72) and at Neumaria during the dry season (0.47), while Merlin and Augustine Camp showed moderate risk (0.13–0.25). PB and Manatee were both considered low risk (< 0.10), consistent with the sparse sand fly captures and the absence of CL reports (Fig. 11). Note in Fig. 11, the CERI value for PB (dry season) reflects a single captured specimen (*Ps. panamensis*) and a very low trap success (0.02 flies trap⁻^1^ night⁻^1^) thus the Jeffreys-adjusted proportion (*P*′ = 0.75) was applied lowering the CERI*log* from 0.33 to 0.25. However, with minimal evidence of sustained vector presence in PB, even this estimate should be interpreted with caution.

## Discussion

Previous quantitative investigations of the sand fly fauna in Belize date back to the 1950s, 1960s, and 1970s [8, 9, 12–14, 19], which sampled the same districts but different areas to the current study. During that period, the current sample sites were predominantly mature forest, but which subsequently have been degraded and/or replaced by agriculture and undergone semi-urban development. This study provides a contemporary quantification of sand fly abundance, indices of sand fly and vector diversity and spatiotemporal patterns in these altered habitats, in addition to a relative risk measure (CERI) of human-sand fly potential exposure.

### Species detection

Twenty of the 28 sand fly species recorded in Belize to date [77] were captured during this study, including two species newly recorded in the country, a single male specimen of each *Lu. longipalpis* and *Pa. soccula*. Collections were dominated by *Da. deleoni* and *Ty. triramula*, followed by *Pi. ovallesi*, *Br. mesai*, and *Ps. panamensis*, which together accounted for > 90% of all captures by CDC light traps and BG-Sentinel traps. The fewer HLC collections yielded a more restricted assemblage dominated by *Ps. panamensis*, *Pi. ovallesi*, *Ty. triramula* and *Da. deleoni*. In the non-HLC collections, *Ty. triramula* and *Da. deleoni* were more dominant than in HLCs (Table 3).

### Vectors

Amongst the species collected, *Ps. panamensis, Pi. ovallesi*, *Bi. olmeca olmeca*, *Pa. shannoni*, *Lu. cruciata, Ny. ylephiletor* and *Lu. longipalpis* are proven vectors of *Leishmania* spp. parasites of the subgenera *Leishmania* or *Viannia* across Central and South America [1, 7, 16–18, 47, 48, 50, 51]. The single *Lu. longipalpis* specimen captured in this study was male, confirming the presence of the species in Belize but does not prove local transmission as only female sand flies transmit *Leishmania* parasites [78]. *Da. deleoni* and *Pa. undulata* have been reported to support natural infections with *L. (L.) mexicana* in Mexico [47], and *Ty. triramula* to support *L. (V.) naiffi* infections in Panama [52], thus they are considered suspect vectors in Belize.

During the historical entomological surveys, Disney (1968), Williams (1965; 1970), and Lainson & Strangways-Dixon (1963; 1966) documented flagellate infections in eight sand fly species, seven of which were captured in the current study. Identification of the *Leishmania* species was attempted using the standard techniques available at the time, including microscopy, live culture, and hamster inoculation. However, infections were only successfully confirmed for *Bi. olmeca olmeca* identified as *L. (L.) mexicana* (Table S6) [8, 11, 13, 14, 48]. In light of the multiple criteria established to incriminate sand fly vectors [79, 80], the historical claims of vectorial capacity need to be interpreted with caution. Here, we consider sand fly species with reported flagellate infections in the absence of confirmed *Leishmania* identification as potential or suspect vectors rather than confirmed vectors.

The current HLCs comprised dominant sand fly species including *Ps. panamensis*, *Pi. ovallesi*, *Da. deleoni*, and *Ty. triramula*, all documented to support natural infections of *Leishmania* as mentioned, and here assumed to have been attracted to host odours in the HLC tent traps, thereby further indicating their potential role in sand fly–human transmission in Belize. The relative vectorial capacity of the later two species is yet to be determined, however their presence appears persistent having been documented in historical HLCs conducted in the Cayo District in Belize by Williams (1970) [14]. The historical and current detection of *Da. deleoni* and *Ty. triramula* in Belize HLCs is of particular interest as they are seldom dominant in HLCs in neighbouring Guatemala or Mexico [81–83] where they are out-numbered by *Ps. panamensis* and *Pi. ovallesi*; the former is a proven vector of *L.* (*V*.) *panamensis*, *L.* (*V*.) *braziliensis, L. amazonensis* and *L.* (*L*.) *mexicana* [16, 50, 84], the latter a vector of *L.* (*V*.) *braziliensis* and *L.* (*L*.) *mexicana* [17, 18, 85, 86].

Other Belizean sand fly species previously reported with natural flagellate infections [14] include *Da. permira* and *Micropygomyia trinidadensis*, both captured during this study, and *Da. steatopyga* which was not, suggesting that additional putative vectors may occur at low abundance or outside the habitats and seasons of the current sample.

### Geographical distributions of vectors

The consistent detection of vectors *Ps. panamensis*, *Pi. ovallesi*, *Bi. olmeca olmeca*, *Pa. shannoni*, and *Lu. cruciata*, across multiple seasons indicates the potential for sustained human-vector exposure. In absolute numbers, the most abundant vector species were *Ps. panamensis* in the wet season and *Pi ovallesi* in the dry season.

Analyses of site-specific species guilds revealed Busby Camp (wet season) and Neumaria (dry season) to be sites with the greatest proportions of vector species (80–94% and 41–47%), respectively (Fig. 8A, B). However, the dominant species of vector differed between sites in some cases: *Pi. ovallesi* was super dominant but only present in two sites (Merlin & Neumaria); *Ps. panamensis* was dominant and present in all sites including PB; while *Bi. olmeca* (Merlin, NM and Augustine)*, Lu. cruciata* (Manatee, NM and Augustine) and* Pa. shannoni* (Marlin, NM and Busby) were present in three sites, but were not dominant.

Vector dominance patterns tended to mirror the associated habitat complexity, with intact mature forest cover associated with greater vector dominance compared to more open habitats such as pine-savannah (Tables 4, 5, 6, 7; Fig. 8A, B). Shaded and humid forest habitats likely favour sand fly breeding and survival, whereas exposed and drier conditions in open habitats like pine savannah, may be more suitable to some species [87]. The abundance and compositions of sand fly blood sources, thought to be predominantly rodents [8, 11, 13, 20, 48, 88, 89], likely differ between habitat types.

### Composite Exposure Risk Index (CERI)

In pursuit of identifying areas of human-sand fly exposure leading to clinical CL disease, the entomological sampling sites were selected in military JTAs where CL incidence amongst visiting troops is systematically recorded thereby providing the opportunity to estimate the geographical risk of human-sand fly infections. The selected sampling sites are where visiting troops typically spend six weeks undergoing field training, nocturnally active or sleeping in exposed conditions, before returning to the UK soon after and where the absence of sand flies precludes transmission. Subsequent clinical and/or molecular diagnosis of CL cases, typically 1–22 weeks post JTA deployment [25], are recorded in the DMICP military database [MOD restricted data], and can be confidently allocated to the respective JTA sampling areas (Table S5).

The calculations of the CERI integrates the CL incidence data, site-specific vector species abundance records, and the fraction of sand fly guilds that are confirmed vectors. The resulting index values accurately reflected the vector dominance patterns already mentioned, ranking Busby Camp and Neumaria as the highest risk areas during wet and dry seasons, respectively, with consistently medium risk at Merlin and Augustine Camp, and low risk at PB and Manatee (Fig. 11). The CERI tool parallels risk-mapping frameworks used in Brazil and Colombia [90–92], employing spatial and spatiotemporal analyses to identify high and low-risk areas. These methodologies are a valuable starting point to guide vector/infection surveillance and for allocation of resources; in the current context, to select JTAs in military health planning. The results will also be of particular relevance to forest guards, loggers and tourists alike.

### Spatiotemporal patterns and seasonality

Marked spatial structuring was evident across the six sampling sites, despite all sites being located within preserved forest areas. Neumaria accounted for 62% of all captures (Table 2), with consistently higher trapping success across consecutive wet and dry seasons (Table 2; Fig. 5), and significantly higher captures than all other sites. This pattern suggests substantial site-level variation in sand fly abundance, although the specific ecological drivers of this variation were not directly assessed in the present study. Species diversity and evenness indices were highest in Neumaria and Augustine Camp and lowest at PB (Fig. 9; Tables 4, 5, 6, 7), further indicating differences in local sand fly community structure across sampling sites. *Da. deleoni* and *Ty. triramula* were the most abundant species in all four sampling sites where they were detected, in contrast *Ps. panamensis* and *Pa. carpenter* occurred across ≥80% of samples sites but were not the most abundant species in the sites. Further ecological assessment would be required to determine whether specific habitat preferences were significant drivers of species distributions.

The lower trapping success at Busby Camp, Augustine and Manatee, despite superficially similar vegetation cover, suggests that micro-scale variations in soil moisture, host abundance, or anthropogenic activity, may drive local suppression of sand flies; nonetheless, competent vectors were present in these sites. The coastal zones of PB and Manatee consistently yielded very few sand fly specimens. Habitat-linked gradients in sand fly species diversity and abundance is documented to increase with forest continuity and decline in disturbed or urban habitats in Yucatán Mexico, Panama and Brazil [93–95]. The same appears to be the case in this region of Belize.

*Lu. longipalpis* is often associated with peridomestic, disturbed and anthropogenically modified environments, and has been reported to expand along newly constructed road corridors in Brazil, consistent with disturbance-associated dispersal [96, 97]. However, the species also shows broad ecological plasticity and has been recorded in a range of habitats, including caves [98]. In the present study, the single *Lu. longipalpis* specimen was collected at Merlin, an area dominated by pine, savannah and secondary forest, near temporary compounds used by road-construction workers associated with an ongoing logging road development project, where dogs were also present. Although no conclusion can be drawn from a single male specimen, its occurrence at this site may reflect the species’ capacity to persist in heterogeneous landscapes where forested, disturbed and peridomestic conditions overlap, [99–101]. This novel record extends the species known distribution northwards from endemic foci in Honduras and adjacent Guatemala [71, 78, 79] and from Mexico [102].

Sand fly diversity was generally higher in the dry seasons, associated with reduced species dominance and more balanced species representation (Fig. 9; Tables 4, 5, 6, 7). Although the core community structure remained consistent across years, species abundance and richness fluctuated significantly with season and year, peaking in the dry season of Year 1 and falling significantly by ~ 72% in Year 2. However, analyses also indicated an inter-annual reversal in seasonal peaks, with wet-season catches ~2.5-fold higher than dry-season catches in Year 2. Inter-annual differences in seasonality may also have been influenced by episodic extreme-weather events. For example, Belize experienced tropical-storm activity associated with heavy rainfall and flooding in 2024 [103] during the trapping period, which we speculate would disrupt breeding sites and sand fly catches, but our data do not support this proposition as sand fly relative abundance was significantly higher in the wet compared to dry season in that year. However, species richness was significantly lower in the wet compared to dry season (Table S4). Thus, while dominant taxa persisted, their abundance was seasonally modulated, possibly by rainfall intensity and microclimatic shifts, which may indicate the presence of microclimatic buffering in forested habitats or the creation of artificial breeding niches through anthropogenic habitat degradation [104, 105]. Such inversions in seasonal peaks were observed in the current study for *Da. deleoni* and *Ty triramula*, where the former peaked during the rainy season and the latter peaked during the dry season; *Ps. panamensis* and *Pi ovallesi* showed similar patterns, respectively (Tables 4, 5, 6, 7).

### Study limitations and future directions

The study had limitations. The use of CDC light traps and HLCs may have biased the results towards more phototaxic and anthropophilic species, respectively, potentially under-representing more zoophilic, less phototaxic or rare taxa. HLCs primarily attract blood-seeking females to host odour cues and body temperature, and therefore may indicate a species propensity towards anthropophily, but not least opportunistic feeding on available human blood sources [106, 107]. By comparison, CDC light traps rely predominantly on phototaxis and can capture both males and females in different physiological and gonotrophic states [106, 108].

Seven sand fly species not collected during this study (Table S6), but previously recorded in the Cayo District, were *Da delpozoi*, *Da. steatopyga*, *Br. hamata, Pa. dasymera* and *Mi. cayennensis*, the latter two recorded also in Orange Walk District, and *Br. hamata* and *M. cayennensis* also recorded in Toledo District [13, 14, 20, 42]. Records of *Lu. manciola* and *Pa. aclydifera* are restricted to Toledo District, the most southern district in Belize (Table S6), suggesting that perhaps in total only ~ 26 species occur in north-west and central-east Belize as suggested by the The ACE and Chao1 estimators. Future deployment of additional trap types may aid in capturing these undetected species, assumed to still occur in the region.

To further understand the site- and temporal variations in sand fly abundance, diversity and species richness, environmental (e.g. vegetation compositions and coverage, soil moisture) and microclimate data would provide opportunities for ecological modelling to map habitat suitability for the more common sand fly species in this study. This will be explored in future studies. Related to this, the study was conducted over a relatively short period (two-years sampling) which precluded scrutinising longer-term climatic cycles such a tropical cyclone frequencies and El Niño–Southern Oscillation events that can influence sand fly dynamics [87, 109, 110].

## Conclusions

This study provides insights into the contemporary spatiotemporal abundance and diversity of the sand fly fauna, including known vectors, in north-west and central-east Belize. In doing so, it identified high and low risk biogeographical areas of potential human–vector contact leading to confirmed clinical CL infections in visiting military personnel. During the two-year study, 20 sand fly species were documented, dominated by *Da. deleoni* and *Ty. triramula*, and including at least seven species known to be vectors of human CL. The study confirms the large number of potential vectors of CL in Belize, and also the presence of *Lu. longipalpis*, the important sand fly vector of *L. infantum* causing visceral leishmaniasis, in addition to *Pa soccula*, both species recorded in Belize for the first time.

## Supplementary Information

Additional file1 (DOCX 341 KB)

## Data Availability

All data generated or analysed during this study are included in this published article and its supplementary information files.
