# Supplementary material for: Spatiotemporal distribution, abundance, diversity and risk of human exposure to phlebotomine sand flies, vectors of Leishmania in Belize
Source: Parasit Vectors. 2026 Aug 7;19:406. doi: 10.1186/s13071-026-07594-9 (PMC13599081; doi:10.1186/s13071-026-07594-9)
Supplement: Supplementary file 1 [file 13071_2026_7594_MOESM1_ESM.docx]

**Supporting information (S)**

**Table of content:**

| **S1 Table:** sex ratios by season and site | 2 |
| --- | --- |
| **S2 Table:** Summary of sex-specific trapping success and sex ratios of sand flies across seasons and years | 2 |
| **S3 Table:** Proportion of sand fly vector species by site and season (with 95 % confidence intervals) | 3 |
| **S4 Table:** Multivariate mixed-effects models for trap success [LMM on log(trap+1)] and species richness (Poisson GLMM). | 4 |
| **S5 Table:** Log-transformed case values, adjusted CERI*log*, and 95% confidence intervals across study sites and seasons | 5 |
| **S6 Table:** Known sand fly species in Belize | 6 |
| **S7 Table:** Historic distribution of phlebotomine sand-fly species by location in Belize | 7 |
| **S1 Figure:** Comparison scatter plot of *original vs log-based* CERI values | 8 |

**Table S1.** Sex ratios by season and site

***Sex ratios by season and site***

Females predominated in most collections, accounting for approximately 80–85% of captures in the wet seasons (F:M = 4.4:1 in Year 1; 5.2:1 in Year 2) (Table S2). In the first dry season, captures were nearly balanced (F:M = 0.9:1), whereas in the second dry season females again predominated (F:M = 2.2:1) (Table S2). Overall, sex ratios differed markedly between wet and dry periods (χ² = 875.6, df = 1, P < 0.001) (Table S2). Female predominance was most evident at inland forest sites such as Neumaria and Merlin, whereas the coastal site Manatee showed a more balanced sex ratio alongside lower overall captures (Tables 4–7).

Species-level patterns also varied. Dampfomyia deleoni was consistently female-biased at Neumaria in both wet seasons (Year 1: F:M = 1077:68 [15.8:1]; Year 2: 911:78 [11.7:1]). In contrast, Pintomyia ovallesi showed a dry-season shift at Neumaria from male-biased in Year 1 (F:M = 149:466 [0.32:1]) to female-biased in Year 2 (120:48 [2.5:1]). Brumptomyia mesai similarly shifted from male-biased in Dry Year 1 (138:293 [0.47:1]) to strongly female-biased in Dry Year 2 (70:11 [6.4:1]) (Tables 4–7).

**Table S2.** Summary of sex-specific trapping success and sex ratios of sand flies across seasons and years

| **Season** | **Year** | **Males** | **Females** | **Total** | **F:M ratio** | **Statistical comparison** |
| --- | --- | --- | --- | --- | --- | --- |
| Wet | 1 | 397 | 1 746 | 2 143 | 4.41:1 | — |
|  | 2 | 297 | 1 550 | 1 847 | 5.22:1 | — |
| Dry | 1 | 1 606 | 1 439 | 3 045 | 0.90:1 | — |
|  | 2 | 197 | 432 | 629 | 2.19:1 | — |
| Overall (all seasons combined) | — | — | — | — | — | t = 1.78, df = 3, p = 0.17 |
| Seasonal difference (wet vs dry) | — | — | — | — | — | χ² (1) = 875.56, p < 0.001 |

Female-to-male ratio by year is 1.6:1 in year 1 and 4:1 in year 2

**Table S3**. Proportion of sand fly vector species by site and season (with 95 % confidence intervals)

| **Season** | **Site** | **Vector total** | **Total captured** | **Proportion** | **CI_lower** | **CI_upper** |
| --- | --- | --- | --- | --- | --- | --- |
| Wet Y1 | Price Barracks | 0 | 0 | NA | NA | NA |
|  | Manatee | 0 | 5 | 0 | 0 | 0 |
|  | Busby Camp | 225 | 280 | 0.804 | 0.752 | 0.848 |
|  | Augustine Camp | 29 | 226 | 0.128 | 0.088 | 0.179 |
|  | Merlin | 50 | 232 | 0.216 | 0.164 | 0.274 |
|  | Neumaria | 125 | 1400 | 0.089 | 0.075 | 0.105 |
| Dry Y1 | Price Barracks | 1 | 1 | 1 | 0.05 | 1 |
|  | Manatee | 4 | 630 | 0.006 | 0.002 | 0.016 |
|  | Busby Camp | 7 | 133 | 0.053 | 0.021 | 0.105 |
|  | Augustine Camp | 21 | 221 | 0.095 | 0.06 | 0.142 |
|  | Merlin | 3 | 319 | 0.009 | 0.002 | 0.027 |
|  | Neumaria | 708 | 1741 | 0.407 | 0.383 | 0.43 |
| Wet Y2 | Price Barracks | 0 | 0 | NA | NA | NA |
|  | Manatee | 0 | 108 | 0 | 0 | 0 |
|  | Busby Camp | 212 | 225 | 0.942 | 0.903 | 0.969 |
|  | Augustine Camp | 3 | 182 | 0.016 | 0.003 | 0.047 |
|  | Merlin | 22 | 195 | 0.113 | 0.072 | 0.166 |
|  | Neumaria | 82 | 1137 | 0.072 | 0.058 | 0.089 |
| Dry Y2 | Price Barracks | 0 | 0 | NA | NA | NA |
|  | Manatee | 0 | 2 | 0 | 0 | 0 |
|  | Busby Camp | 2 | 47 | 0.043 | 0.005 | 0.145 |
|  | Augustine Camp | 13 | 71 | 0.183 | 0.101 | 0.293 |
|  | Merlin | 2 | 42 | 0.048 | 0.006 | 0.162 |
|  | Neumaria | 217 | 467 | 0.465 | 0.419 | 0.511 |

The table shows the proportion of captured specimens identified as known or suspected vector species across six sites over two wet and two dry seasons. Confidence intervals were calculated using the exact Clopper–Pearson method. “NA” indicates sites or seasons where captures were insufficient to estimate proportions reliably.

**Table S4.** Multivariate mixed-effects models for trap success [LMM on log(trap+1)] and species richness (Poisson GLMM).

| **Model / Effect** | **β (Estimate)** | **SE** | **z / t** | **p-value** | **95% CI (β)** | **Back-transform** |
| --- | --- | --- | --- | --- | --- | --- |
| **Trap success (LMM on log(trap+1))** | | | | | | |
| Intercept (Dry, Year 1) | 2.296 | 0.517 | 4.44 | <0.001 | 1.283 to 3.308 | Baseline E[log(trap+1)] |
| Wet (vs Dry) | –0.688 | 0.373 | –1.85 | 0.065 | –1.419 to 0.042 | **×0.50** (–50%) in Year 1 |
| Year 2 (vs Year 1) | –1.278 | 0.373 | –3.43 | **0.001** | –2.008 to –0.547 | **×0.28** (–72%) |
| Wet × Year 2 | 1.604 | 0.527 | 3.04 | **0.002** | 0.571 to 2.637 | **×4.97** change of wet effect |
| Site random variance | **1.185** | — | — | — | — | (SD ≈ 1.088) |
| Residual variance | **0.417** | — | — | — | — | (SD ≈ 0.646) |
| LR test vs linear model | — | — | — | **0.0001** | — | Random effect needed |
| ICC (site) | **0.740** | 0.153 | — | — | 0.375 to 0.931 | Strong clustering by site |
| **Species richness (Poisson GLMM)** | | | | | | |
| Intercept (Dry, Year 1) | 1.603 | 0.415 | 3.86 | <0.001 | 0.790 to 2.417 | Baseline mean richness μ = e¹·⁶⁰³ ≈ 4.97 spp. |
| Wet (vs Dry) | –0.623 | 0.264 | –2.36 | **0.018** | –1.141 to –0.105 | **IRR = e⁻⁰·⁶²³ ≈ 0.54 (≈ 46% lower richness)** |
| Year 2 (vs Year 1) | –0.495 | 0.254 | –1.95 | 0.051 | –0.992 to 0.003 | IRR ≈ 0.61 (≈ 39% lower, borderline significance) |
| Wet × Year 2 | 0.294 | 0.407 | 0.72 | 0.470 | –0.503 to 1.091 | IRR ≈ 1.34 (no evidence of interaction) |
| Site random variance | **0.816** | — | — | — | 0.182 to 3.66 | SD ≈ 0.903 |
| LR test vs fixed Poisson | — | — | — | **< 0.001** | — | Random site effect strongly supported |

Fixed effects shown with estimates (β), standard errors (SE), test statistics (z/t), p-values, and back-transformed effects (IRR or multiplicative change). Random-effect variance reported for site; for the LMM, ICC is also reported. CI confidence interval, SD standard deviation,

**Table S5.** Log-transformed case values, adjusted CERI*log*, and 95% confidence intervals across study sites and seasons

| **Season** | **Site** | **Proportion of vector species (P)** | **Mean vector species trapping success (flies per trap per night)** | | **Cases^b^** | **log(Cases + 0.5)** | **Normalised C′ (log-scale)** | **CERI*log*** | **95% CI for log(Cases + 0.5)*** | **95 % CI (approx.)** | **Risk category^β^** |
| --- | --- | --- | --- | --- | --- | --- | --- | --- | --- | --- | --- |
|  |  |  | **F** | **F_norm_** |  |  |  |  |  |  |  |
| Wet^a^ | Price Barracks | 0.00 | 0.00 | 0.00 | 0 | -0.69 | 0.00 | 0.00 | [-0.69, -0.69] † | 0.00-0.00 | Low |
|  | Manatee | 0.00 | 0.00 | 0.00 | 0 | -0.69 | 0.00 | 0.00 | [-0.69, -0.69] † | 0.00-0.00 | Low |
|  | Busby Camp | 0.87 | 10.40 | 0.297 | 35 | 3.58 | 1.00 | 0.72 | [2.96, 4.21] | 0.63-0.81 | High |
|  | Augustine Camp | 0.08 | 0.38 | 0.011 | 9 | 2.35 | 0.65 | 0.25 | [1.72, 2.98] | 0.17-0.33 | Medium |
|  | Merlin | 0.17 | 0.86 | 0.025 | 3 | 1.39 | 0.38 | 0.19 | [0.76, 2.01] | 0.12-0.27 | Medium |
|  | Neumaria | 0.08 | 2.46 | 0.070 | 0 | -0.69 | 0.00 | 0.05 | [-0.69, -0.69] † | 0.00-0.12 | Low |
| Dry^a^ | Price Barracks (Jeffreys adj.) | 0.75 | 0.01 | 0.00 | 0 | -0.69 | 0.00 | 0.25^α^ | [-0.69, -0.69] † | 0.17-0.34 | Medium |
|  | Manatee | 0.01 | 0.07 | 0.002 | 0 | -0.69 | 0.00 | 0.00 | [-0.69, -0.69] † | 0.00-0.00 | Low |
|  | Busby Camp | 0.05 | 0.16 | 0.005 | 0 | -0.69 | 0.00 | 0.02 | [-0.69, -0.69] † | 0.00-0.07 | Low |
|  | Augustine Camp | 0.12 | 0.61 | 0.017 | 2 | 0.92 | 0.26 | 0.13 | [0.29, 1.55] | 0.06-0.20 | Medium |
|  | Merlin | 0.01 | 12.68 | 0.363 | 0 | -0.69 | 0.00 | 0.12 | [-0.69, -0.69] † | 0.05-0.19 | Medium |
|  | Neumaria | 0.42 | 34.96 | 1.00 | 0 | -0.69 | 0.00 | 0.47 | [-0.69, -0.69] † | 0.38-0.56 | High |

a Average female sand fly trap rates per site (combined for both years)

b 49 CL cases reported in 2100 troops who deployed from 2019 to 2022 [1], plus an additional 0 CL cases reported in 1200 troops deployed from 2023 to 2025 [MOD restricted data].

α Jeffreys correction (Beta 0.5, 0.5 prior). At *Price Barracks (dry season)*, only one specimen was captured, a vector species, yielding an apparent 100 % vector proportion. To avoid inflation from this single observation, a Jeffreys correction (Beta 0.5, 0.5 prior) was applied, producing an adjusted P′ = 0.75 and correspondingly lower CERIₗₒg (0.25 vs 0.33). This stabilises the estimate while retaining the site’s inclusion for completeness.

β CERI score categorised as follows: Low risk (<0.10, light blue), Medium risk (0.10 – 0.30, orange-peach), and High risk (>0.30, red) [2]. CL or cutaneous leishmaniasis, CERI or composite exposure risk index.

* 95% CI approximated assuming ±1.96 × standard deviation around the log(C + 0.5) mean per site/season.

† No variance because all zero cases recorded.

Notes: The CERI*log* values were computed as the mean of normalised P′, F′, and log-transformed C′.

**Table S6.** Known sand fly species in Belize

| **Former nomenclature** | **New nomenclature^a^** | **District** | **Flagellates detected in Belize (*Leishmania* species)** | **Suspected species elsewhere in the Americas (*Leishmania* species)** |
| --- | --- | --- | --- | --- |
| *Lutzomyia panamensis* (Shannon, 1926) | *Psychodopygus panamensis*^1^ | Cayo | Yes (unknown) |  |
| *Lu. geniculata* (Mangabeira, 1941) | *Psychodopygus geniculatus* | Cayo | *No* |  |
| *Lu. bispinosa* (Fairchild & Hertig, 1951) | *Psychodopygus bispinosus*^2^ | Cayo, Orange Walk, Toledo | *No* | Guatemala (*Leishmania* sp*.*) |
| *Lu. disneyi* (Williams, 1987) | *Dampfomyia disneyi* | Cayo, Toledo | *No* |  |
| *Lu. delpozoi* (Vargas & Dõ ́az-Na ́jera, 1953) | *Dampfomyia delpozoi^*^* | Cayo | *No* |  |
| *Lu. deleoni* (Fairchild and Hertig, 1947) | *Dampfomyia deleoni*^2^ | Cayo, Toledo | *No* | Mexico (*L*. [*L*.] *mexicana*) |
| *Lu. serrana* (Damasceno & Arouck, 1949) | *Pintomyia serrana* | Toledo | *No* |  |
| *Lu. permira* (Fairchild & Hertig, 1956) | *Dampfomyia permira*^2^ | Cayo | Yes (unknown) |  |
| *Lu. trinidadensis* (Newstead, 1922) | *Micropygomyia trinidadensis*^2^ | Cayo | Yes (unknown) | Peru (*Leishmania sp.*) |
| *Lu. olmeca* (Vargas & Dõ ́az-Na ́jera, 1959) | *Bichromomyia olmeca olmeca*^1^ | Cayo, Orange Walk, Toledo | *L. (L) mexicana* |  |
| *Lu. ylephiletor* (Fairchild & Hertig, 1952) | *Nyssomyia ylephiletor*^1^ | Cayo, Orange Walk, Toledo | *No* |  |
| *Lu. cruciata* (Coquillett, 1907) | *Lu. cruciata*^1^ | Cayo, Toledo | Yes (unknown) |  |
| *Lu. ovallesi* (Ortiz, 1952) | *Pintomyia ovallesi*^1^ | Cayo, Toledo | Yes (unknown) |  |
| *Lu. carpenteri* (Fairchild & Hertig, 1953) | *Psathyromyia carpenteri*^2^ | Cayo, Toledo | *No* | Mexico (*Leishmania sp.*) |
| *Lu. aclydifera* (Fairchild & Hertig, 1952) | *Psathyromyia aclydifera^*^*^2^ | Toledo | *No* | Mexico (*Leishmania sp.*) |
| *Lu. shannoni* (Dyar, 1929) | *Psathyromyia shannoni*^1^ | Cayo, Toledo | Yes (unknown) |  |
| *Lu. dasymera* (Fairchild & Hertig, 1961) | *Psathyromyia dasymera^*^* | Cayo, Orange Walk | *No* |  |
| *Lu. cratifer* (Fairchild & Hertig, 1961) | *Psathyromyia cratifer*^2^ | Cayo | *No* | Mexico (*L*. [*L*.] *mexicana*) |
| *Lu. undulata* (Fairchild & Hertig, 1950) | *Psathyromyia undulata*^2^ | Cayo | *No* | Mexico (*L*. [*L*.] *mexicana*) |
| *Lu. manciola* (Ibáñez-Bernal, 2001) | *Lu. manciola^*^* | Toledo | *No* |  |
| *Lu. cayennensis* (Floch & Abonnenc, 1941) | *Micropygomyia cayennensis^*^* ^2^ | Cayo, Orange Walk, Toledo | *No* | Ecuador (*L*. [*L*.]  *mexicana)* |
| *Lu. triramula* (Fairchild & Hertig, 1952) | *Trichopygomyia triramula^2^* | Cayo | *No* | Panama (*Leishmania naiffi*) |
| *Lu. steatopyga* (Fairchild & Hertig, 1958) | *Dampfomyia steatopyga^*^* ^2^ | Cayo | Yes (unknown) |  |
| *Brumptomyia mesai* (Sherlock, 1962) | *Brumptomyia mesai*^2^ | Cayo, Toledo | *No* | Mexico (*Leishmania sp.*) |
| *Brumptomyia hamata* (Fairchild & Hertig 1947) | *Brumptomyia hamata^*^* | Cayo, Toledo | *No* |  |
| *Brumptomyia leopoldoi* (Rodrõguez 1953) | *Brumptomyia leopoldoi*^2^ | Cayo, Toledo | *No* | Ecuador (*Leishmania* sp*. siamensi)* |

a Classification & nomenclature proposed by Galati (2018) [3]. * Seven sand fly species not recorded in this study. ^1^ Proven vector species in the Americas [4–8]

^2^ Suspected vector species [9–17]. Sand fly species reported from Belize were collected in studies by Martins et al. (1978); Williams (1965, 1970); Strangways-Dixon & Lainson (1963, 1964, 1966); Fairchild & Hertig (1959); Lewis & Garnham (1959, 1965); and Disney (1964).

**Table S7.** Historic distribution of phlebotomine sand-fly species by location in Belize

| **Locality (District)** | **Habitat type / Notes** | **Sand-fly species recorded*** | **Primary references** |
| --- | --- | --- | --- |
| Gallon Jug (Orange Walk District) | High-forest chicle/mahogany area; known CL focus | *Ps. panamensis, Pa. shannoni, Lu. cruciata, Pi. ovallesi, Pi. pessoanus, Ps. bispinosus, Ny. ylephiletor, Pa. apicalis* | Williams 1965 [18] |
| Spanish Lookout (Cayo District) | Peridomestic / low-bush area near Mennonite settlement (human CL case) | *Ps. panamensis, Pi. ovallesi, Pa. shannoni, Lu. cruciata, Ps. bispinosus* | Williams 1965 [18] |
| Central Farm (Cayo District) | Low-bush near cleared land; infected rodents, no human cases | *Ps. cruciatus, Ps. panamensis, Pa. shannoni, Pi. ovallesi, Ps. bispinosus* | Lainson & Strangways-Dixon 1963 [19]; Williams 1965 [18] |
| Baking Pot (Belize River valley) (Cayo District) | Medium-bush with dense undergrowth; no human CL cases | *Pi. ovallesi, Ps. panamensis, Ps. cruciatus, Ps. bispinosus, Pa. shannoni* | Williams 1965 [18] |
| Roaring River (Cayo District) | Confirmed CL focus (9 patients, 15 infected rodents) | *Ps. panamensis, Ps. bispinosus, Pi. ovallesi, Pa. shannoni, Lu. cruciata* | Williams 1965 [18]  Disney 1968 [20] |
| Sibun Camp (Cayo District) | Cocoa plantation area; 1 human CL case | *Ps. bispinosus, Ps. panamensis, Pa. shannoni, Pi. ovallesi* | Williams 1965 [18] |
| Chiquibul Road (Cayo District) | High-forest area with infected rodents (L. mexicana) | *Ps. panamensis, Pi. ovallesi, Pa. apicalis, Pa. shannoni* | Lainson & Strangways-Dixon 1964 [21]; Williams 1965 [18] |
| Guacamallo (Cayo District) | High-forest area; no CL cases recorded | *Pi. ovallesi, Ps. panamensis, Pa. shannoni* | Williams 1965 [18] |
| Millionario (Cayo District) | Medium-bush chicle/mahogany camp; historic CL cases (1958) | *Ps. panamensis, Pa. shannoni, Lu. cruciata, Pi. ovallesi, Da. deleoni, Ps. bispinosus* | Williams 1965 [18] |
| Augustine (Cayo District) | Bat cave collection site; no human cases | *Pa. shannoni, Lu. cruciata, Pi. ovallesi* | Williams 1965 [18] |
| Toledo District (Log Yard) | High-forest with sparse undergrowth; no previous records | *Da. deleoni, Pa. apicalis, Pi. ovallesi, Ps. panamensis, Pa. shannoni* | Williams 1965 [18] |

Notes: *Nomenclature updated following Galati [3,22]. Species composition mainly reflects human-bait collections (Williams 1965 [18]) some taxa (e.g. *Da. deleoni*) also recorded by Disney (1966) [23] using rodent-bait traps. Table consolidates all historical data available up to 1970.

**Figure S1**: Comparison scatter plot of *original vs log-based* CERI values

**References**

1.	Rawlings NN, Bailey M, Craig P, Courtenay O. Cutaneous leishmaniasis in British troops following jungle training in Belize: cumulative incidence and potential risk practices [Internet]. medRxiv; 2024 [cited 2024 Mar 12]. p. 2024.01.30.24302002. Available from: https://www.medrxiv.org/content/10.1101/2024.01.30.24302002v1 doi:10.1101/2024.01.30.24302002

2.	Kerr KF, Wang Z, Janes H, McClelland RL, Psaty BM, Pepe MS. Net reclassification indices for evaluating risk prediction instruments: a critical review. Epidemiology. 2014 Jan;25(1):114–21. doi:10.1097/EDE.0000000000000018 PubMed PMID: 24240655; PubMed Central PMCID: PMC3918180.

3.	Galati EAB. Phlebotominae (Diptera, Psychodidae): Classification, Morphology and Terminology of Adults and Identification of American Taxa. In: Rangel EF, Shaw JJ, editors. Brazilian Sand Flies : Biology, Taxonomy, Medical Importance and Control [Internet]. Cham: Springer International Publishing; 2018 [cited 2024 Apr 15]. p. 9–212. Available from: https://doi.org/10.1007/978-3-319-75544-1_2 doi:10.1007/978-3-319-75544-1_2

4.	López M, Erazo D, Hoyos J, León C, Fuya P, Lugo L, Cordovez JM, González C. Measuring spatial co-occurrences of species potentially involved in Leishmania transmission cycles through a predictive and fieldwork approach. Sci Rep. 2021 Mar 24;11(1):6789. doi:10.1038/s41598-021-85763-9

5.	Canché-Pool EB, Panti-May JA, Ruiz-Piña HA, Torres-Castro M, Escobedo-Ortegón FJ, Tamay-Segovia P, Blum-Domínguez S, Torres-Castro JR, Reyes-Novelo E. Cutaneous Leishmaniasis Emergence in Southeastern Mexico: The Case of the State of Yucatan. Trop Med Infect Dis. 2022 Dec 17;7(12):444. doi:10.3390/tropicalmed7120444 PubMed PMID: 36548699; PubMed Central PMCID: PMC9787846.

6.	Lopez Y, Casas-Sanchez A, Arana B, Rizzo N, Duran E, Padilla N, de la Vega A, Bustamante E, Acosta-Serrano Á, Mendizabal-Cabrera R. Insights into the transmission cycle of cutaneous leishmaniasis from an endemic community in rural Guatemala. Current Research in Parasitology & Vector-Borne Diseases. 2025 Jan 1;7:100244. doi:10.1016/j.crpvbd.2025.100244

7.	González C, Rebollar-Téllez EA, Ibáñez-Bernal S, Becker-Fauser I, Martínez-Meyer E, Peterson AT, Sánchez-Cordero V. Current knowledge of Leishmania vectors in Mexico: how geographic distributions of species relate to transmission areas. Am J Trop Med Hyg. 2011 Nov;85(5):839–46. doi:10.4269/ajtmh.2011.10-0452 PubMed PMID: 22049037; PubMed Central PMCID: PMC3205629.

8.	Vadmal GM, Glidden CK, Han BA, Carvalho BM, Castellanos AA, Mordecai EA. Data-driven predictions of potential Leishmania vectors in the Americas. PLoS Negl Trop Dis. 2023 Feb 21;17(2):e0010749. doi:10.1371/journal.pntd.0010749 PubMed PMID: 36809249; PubMed Central PMCID: PMC9983874.

9.	Quiroga C, Cevallos V, Morales D, Baldeón ME, Cárdenas P, Rojas-Silva P, Ponce P. Molecular Identification of Leishmania spp. in Sand Flies (Diptera: Psychodidae, Phlebotominae) From Ecuador. J Med Entomol. 2017 Nov 7;54(6):1704–11. doi:10.1093/jme/tjx122 PubMed PMID: 28981860.

10.	Porter CH, Steurer FJ, Kreutzer RD. Isolation of Leishmania mexicana mexicana from Lutzomyia ylephiletor in Guatemala. Trans R Soc Trop Med Hyg. 1987 Nov 1;81(6):929–30. doi:10.1016/0035-9203(87)90355-5 PubMed PMID: 3503412.

11.	Cañeda-Guzmán IC, de Oca-Aguilar ACM, Miranda-Caballero CI, Grostieta E, Correa-Morales F, Romero-Pérez R, Romero-Contreras FE, Rodríguez-Atanacio JA, Ruiz-Tovar K, Huerta H, Mis-Avila PedroC, Quintanilla-Cedillo MR, Lammoglia-Villagómez MA, Blum-Domínguez S, Tamay-Segovia P, Rojas-Ronquillo R, Sánchez-Montes S, Becker I. Entomological Survey and Leishmania (Leishmania) mexicana Prevalence in Sand Fly Species during an Outbreak of Cutaneous Leishmaniasis in Quintana Roo State, Mexico. Trop Med Infect Dis. 2023 Oct 5;8(10):465. doi:10.3390/tropicalmed8100465 PubMed PMID: 37888593; PubMed Central PMCID: PMC10610947.

12.	Oca-Aguilar ACM de, Fernández-Figueroa EA, López-Ávila KB, Pavón-Méndez MI, Sosa-Bibiano EI, Rebollar-Téllez EA, Palacio-Vargas JA, García-López B, Rangel-Escareño C, Loría-Cervera EN. Abundance and Leishmania infection patterns of the sand fly Psathyromyia cratifer in Southern Mexico. PLOS Neglected Tropical Diseases. 2024 Sep 10;18(9):e0012426. doi:10.1371/journal.pntd.0012426

13.	Montes de Oca-Aguilar AC, Pavón-Mendez MI, López-Ávila KB, Sosa-Bibiano EI, Rebollar-Téllez EA, Palacio-Vargas JA, Fernández-Figueroa EA, Loría-Cervera EN. Biting rhythms and infection rates of anthropophilic sand fly species (Diptera: Phlebotominae) in sites with different land use in southern Mexico. Acta Tropica. 2023 Dec 1;248:107014. doi:10.1016/j.actatropica.2023.107014

14.	Lozano-Sardaneta YN, Sánchez-Montes S, Sánchez-Cordero V, Becker I, Paternina LE. Molecular detection of *Leishmania infantum* in sand flies (Diptera: Psychodidae: Phlebotominae) from Veracruz, Mexico. Acta Tropica. 2020 Jul 1;207:105492. doi:10.1016/j.actatropica.2020.105492

15.	Valdivia HO, De Los Santos MB, Fernandez R, Baldeviano GC, Zorrilla VO, Vera H, Lucas CM, Edgel KA, Lescano AG, Mundal KD, Graf PCF. Natural Leishmania Infection of Lutzomyia auraensis in Madre de Dios, Peru, Detected by a Fluorescence Resonance Energy Transfer–Based Real-Time Polymerase Chain Reaction. Am J Trop Med Hyg. 2012 Sep 5;87(3):511–7. doi:10.4269/ajtmh.2012.11-0708 PubMed PMID: 22802444; PubMed Central PMCID: PMC3435357.

16.	Williams P. Phlebotomine sandflies and leishmaniasis in British honduras (Belize). Transactions of The Royal Society of Tropical Medicine and Hygiene. 1970 Jan 1;64(3):317–63. doi:10.1016/0035-9203(70)90171-9

17.	Rigg CA, Calzada JE, Saldaña A, Perea M, Chaves LF, Valderrama A. Leishmania spp. Infection Rate and Feeding Patterns of Sand Flies (Diptera: Psychodidae) from a Hyperendemic Cutaneous Leishmaniasis Community in Panamá. Am J Trop Med Hyg. 2019 Apr;100(4):798–807. doi:10.4269/ajtmh.17-0628 PubMed PMID: 30793681; PubMed Central PMCID: PMC6447106.

18.	Williams P. Observations on the phlebotomine sandflies of British Honduras. Ann Trop Med Parasitol. 1965 Dec;59(4):393–404. doi:10.1080/00034983.1965.11686325 PubMed PMID: 5862607.

19.	Lainson R, Strangways-Dixon J. Leishmania mexicana: The epidemiology of dermal leishmaniasis in British Honduras. Transactions of the Royal Society of Tropical Medicine and Hygiene. 1963 Jul 1;57(4):242–65. doi:10.1016/0035-9203(63)90182-2

20.	Disney RHL. The discovery of the vector of Leishmania mexicana. Transactions of The Royal Society of Tropical Medicine and Hygiene. 1968 Jan 1;62(3):457. doi:10.1016/0035-9203(68)90103-X

21.	Lainson R, Strangways-Dixon J. The epidemiology of dermal leishmaniasis in British Honduras: Part II. Reservoir-hosts of Leishmania mexicana among the forest rodents. Transactions of The Royal Society of Tropical Medicine and Hygiene. 1964 Mar 1;58(2):136–53. doi:10.1016/0035-9203(64)90003-3

22.	Galati EAB, Galvis-Ovallos F, Lawyer P, Léger N, Depaquit J. An illustrated guide for characters and terminology used in descriptions of Phlebotominae (Diptera, Psychodidae). Parasite. 2017 Jul 21;24:26. doi:10.1051/parasite/2017027 PubMed PMID: 28730992.

23.	Disney RHL. A trap for Phlebotomine sandflies attracted to rats. Bulletin of Entomological Research. 1966 Jun;56(3):445–51. doi:10.1017/S0007485300056510
